# Nonstoichiometry Promoted Solventless Recrystallization of a Thick and Compact CsPbBr_3_ Film for Real‐Time Dynamic X‐Ray Imaging

**DOI:** 10.1002/advs.202407314

**Published:** 2024-10-21

**Authors:** Jian Wang, Shanshan Yu, Handong Jin, Yu Li, Kai Zhang, David Lee Phillips, Shihe Yang

**Affiliations:** ^1^ Institute of Biomedical Engineering Shenzhen Bay Laboratory Shenzhen Guangdong 518107 China; ^2^ Guangdong Provincial Key Lab of Nano‐Micro Materials Research School of Advanced Materials Shenzhen Graduate School Peking University Shenzhen Guangdong 518055 China; ^3^ Department of Chemistry The University of Hong Kong Hong Kong SAR 999077 China

**Keywords:** aerosol–liquid–solid, all‐inorganic perovskite, Ostwald ripening, real‐time dynamic imaging, X‐ray detector

## Abstract

Inorganic CsPbBr_3_ perovskite emerges as a promising material for the development of next‐generation X‐ray detectors. However, the formation of a high‐quality thick film of CsPbBr_3_ has been challenging due to the low solubility of its precursor and its high melting point. To address this limitation, a nonstoichiometry approach is taken that allows lower‐temperature crystallization of the target perovskite under the solventless condition. This approach capitalizes on the presence of excess volatile PbBr_2_ within the CsPbBr_3_ film, which induces melting point depression and promotes recrystallization of CsPbBr_3_ at a temperature much lower than its melting point concomitant with the escape of PbBr_2_. Consequently, thick and compact films of CsPbBr_3_ are formed with grains ten times larger than those in the pristine films. The resulting X‐ray detector exhibits a remarkable sensitivity of 4.2 × 10^4^ µC Gy_air_^−1^ cm^−2^ and a low detection limit of 136 nGy_air_ s^−1^, along with exceptional operational stability. Notably, the CsPbBr_3_‐based flat‐panel detector achieves a high resolution of 0.65 lp pix^−1^ and the first demonstration of real‐time dynamic X‐ray imaging for perovskite‐based devices.

## Graphical abstract

Thick and compact CsPbBr_3_ film is prepared with a nonstoichiometric approach by capitalizing on the presence of excess volatile PbBr_2_ that induces melting point depression and promotes recrystallization of CsPbBr_3_ at a temperature much lower than its melting point. The corresponding flat‐panel X‐ray detector realizes high‐quality real‐time dynamic imaging without any motion artifact, suggesting its high application potential.

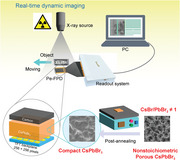

## 1 Introduction

X‐ray detection has become an indispensable technology in wide domains, encompassing medical radiography, security screening, crystal structure analysis, etc. In general, X‐ray detectors can be divided into indirect‐conversion detectors and direct‐conversion detectors depending on whether the detection is via a scintillation step or by collecting the directly generated electrons. Despite their high sensitivity, indirect‐conversion X‐ray detectors suffer from low spatial resolution, likely giving way to direct‐conversion detectors with high untapped potential.^[^ ^1^, ^2^ ^]^ In recent years, metal halide perovskites have garnered increasing attention as promising materials for next‐generation radiation detectors due to their exceptional optoelectronic properties, including high atomic number, substantial mobility‐lifetime product, and robust defect tolerance.^[^ ^3^, ^4^, ^5^, ^6^, ^7^ ^]^ In comparison to their organic‐inorganic hybrid counterparts, all‐inorganic perovskites like CsPbBr_3_ demonstrate superior chemical and structural stability, particularly under high ionizing energy conditions.^[^ ^8^, ^9^ ^]^ However, the solubility‐limited low concentration of inorganic CsPbBr_3_ perovskite precursors in solutions poses thorny challenges in fabricating thick, dense, and large‐grain CsPbBr_3_ films on substrates to enhance the radiation absorption and charge carrier transport properties. Various methods have been explored to address this challenge and enable the absorption of radiation energy, such as single crystal growth^[^ ^2^, ^9^ ^]^ and wafer preparation.^[^ ^10^ ^]^ However, integrating single crystals and wafers to thin‐film transistor (TFT) arrays proves difficult and costly, if not impossible.^[^ ^6^, ^10^, ^11^ ^]^ Recently, melt processing has been employed to produce thick quasi‐monocrystalline CsPbBr_3_ films with excellent electro‐optical properties.^[^ ^12^, ^13^ ^]^ For example, Pan et al. melted CsPbBr_3_ powders on an FTO substrate at 600 °C in a furnace and subsequently covered them with quartz to form a thick film, which allowed to achieve a sensitivity of 55 684 µC Gy_air_^−1^ cm^−2^. However, one thing that bothers is that this method requires the operating temperatures to be higher than the material's melting point (≈560 °C), and thus leads to increased energy consumption and little chance to integrate with silicon‐based readout electronics.

Another challenge associated with preparing the thick perovskite film for flat‐panel detectors (FPDs) is how to make the film scalable because detector areas of 24 × 30 cm^2^ and 30 × 40 cm^2^ are generally required for generic mammography and angiography, respectively.^[^ ^14^ ^]^ The film uniformity is also critical if one is to obtain a high‐fidelity X‐ray image, necessitating precise control over the film thickness, charge carrier transport, as well as bonding with each pixel electrode. Compared with single crystals that are difficult to upscale, polycrystalline films show great superiority because of their capability of deposition on large‐area TFT or complementary metal‐oxide‐semiconductor (CMOS) arrays. Blade or screen coating methods have been adopted to fabricate the perovskite‐based FPDs (Pe‐FPDs).^[^ ^3^, ^15^, ^16^, ^17^ ^]^ However, voids and pinholes are inevitable because the residual solvent in the thick film must be evaporated from within, leading to inefficient X‐ray absorption, nonuniform attachment to the bottom layer, rough surface, and high defect densities. Therefore, developing a method for preparing thick, compact, scalable CsPbBr_3_ perovskite films at a moderate temperature is of great significance for fabricating high‐performance direct‐conversion X‐ray FPDs and remains a great challenge now.

Post‐treatment involving solvent or salt assistance is an effective strategy for mitigating pin holes and augmenting grain sizes in perovskite films through the Ostwald ripening mechanism.^[^ ^18^, ^19^ ^]^ Compared to solvent annealing in, for instance, DMF or DMSO, salt treatment offers enhanced efficacy and simplicity due to more controllable solid reactions.^[^ ^20^ ^]^ Given the soft‐matter characteristics of halide perovskite, salt vapor can readily interact with the perovskite bulk grains to promote the fluidization of grain surfaces and their subsequent amalgamation into substantially larger ones.^[^ ^19^ ^]^

Previously, we introduced a post‐ripening aerosol–liquid–solid (PRALS) technique to produce phase‐pure CsPbBr_3_ films featuring enlarged grain sizes.^[^ ^21^ ^]^ A brief annealing step at 400 °C facilitated the melting and recrystallization of small grains into larger ones via the Ostwald ripening process, but the detailed mechanism was left unexplained and warrants further investigation for enhancing the film growth controllability.^[^ ^22^ ^]^ In this work, we demonstrate that the excess PbBr_2_ plays a critical role in inducing the under‐temperature melting and recrystallization of CsPbBr_3_ into a high‐quality film at a much lower temperature. By increasing the PbBr_2_ to CsPbBr_3_ ratio in the pristine precursor film, the melting occurs at a much lower temperature, leading to recrystallization and Ostwald ripening of the CsPbBr_3_ film concomitant with the sublimation of PbBr_2_. This enables us to controllably and reproducibly prepare thick, compact CsPbBr_3_ films with large grain sizes and in excellent contact with the substrate at a relatively low temperature. The prototype X‐ray detector based on the compact CsPbBr_3_ film exhibits a highly sensitive X‐ray response. The fabricated FPD achieves a spatial resolution of 0.65 lp pix^−1^ at a modulation transfer function (MTF) of 0.2, which is the highest among all the reported Pe‐FPDs. Finally, for the first time, real‐time dynamic X‐ray imaging using the Pe‐FPDs was demonstrated.

## 2 Results and Discussion

### 2.1 Fabrication and Characterization of CsPbBr_3_ Films

**Figure 1:**
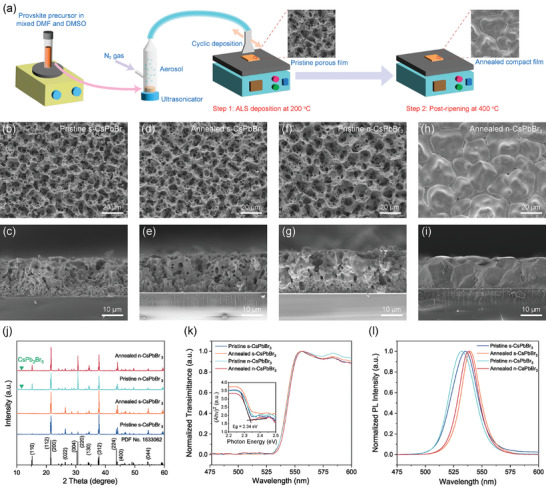
Fabrication and Characterization of CsPbBr_3_ films. a) Schematic illustrating the fabrication process of CsPbBr_3_ films via a two‐step post‐ripening aerosol–liquid–solid method. b–i) Top‐view and cross‐sectional SEM images of the s‐CsPbBr_3_ and n‐CsPbBr_3_ films before and after annealing treatment. j–l) XRD patterns (j), transmittance spectra (k), and PL spectra (l) of s‐CsPbBr_3_ and n‐CsPbBr_3_ films before and after annealing, respectively.

We prepared the pristine CsPbBr_3_ films with different stoichiometries by tuning the CsBr/PbBr_2_ ratio (1 and 0.92) in precursor solutions. Because of the low solubility of CsBr in mixed DMF/DMSO solvent, the concentrations of all the precursor solutions are as low as 0.04 m. Then the CsPbBr_3_ film was deposited on the FTO substrate by a home‐made ALS setup at 200 °C in ambient condition, followed by annealing at 400 °C for another 5 min,^[^ ^21^, ^23^, ^24^, ^25^, ^26^, ^27^, ^28^, ^29^ ^]^ as schematically illustrated in **Figure** **1**a. The film thickness was controlled by adjusting the number of depositions. As shown in Figure 1b–i, before annealing, both the pristine stoichiometric CsPbBr_3_ (CsBr/PbBr_2_ ratio of 1, termed as s‐CsPbBr_3_) film and non‐stoichiometric CsPbBr_3_ film (CsBr/PbBr_2_ ratio of 0.92, termed as n‐CsPbBr_3_) show a similarly porous morphology made of small grains after ALS deposition. This porous nature of the films is caused by the relatively high solvent evaporation rate at 200 °C during deposition, and the perovskite would nucleate from the top part of the liquid zone, resulting in the non‐continuous growth of perovskite along the vertical direction and the formation of small grains and voids inside the film.^[^ ^25^ ^]^ Impressively, after annealing at 400 °C, a temperature much lower than the melting point of CsPbBr_3_ (≈560 °C), the n‐CsPbBr_3_ film transforms into a more compact structure with a little decrease in film thickness, and the grain sizes are significantly enlarged by more than ten times to over 10 µm. In contrast, the morphology and thickness of the s‐CsPbBr_3_ film remain almost unchanged after annealing. From the cross‐sectional SEM image (Figure 1i), we can observe that the annealed n‐CsPbBr_3_ film exhibits a more densified structure without any exfoliation from the substrate, suggesting excellent adhesion between the perovskite film and the substrate.

The X‐ray diffraction patterns (Figure 1j) reveal that the s‐CsPbBr_3_ and n‐CsPbBr_3_ can be assigned to the orthorhombic phase (PDF Card No.: 1 533 062). An impurity phase, which arises from CsPb_2_Br_5_, is observed in the n‐CsPbBr_3_ at ≈11.6 degree. The formation of CsPb_2_Br_5_ impurity during the deposition is caused by the higher ratio of PbBr_2_ over that of CsBr in the precursor solution, which easily forms the Pb‐rich phase CsPb_2_Br_5_ during the deposition. This impurity peak decreases after annealing at 400 °C due to the decomposition of CsPb_2_Br_5_ into CsPbBr_3_ and PbBr_2_, as well as the easy sublimation of PbBr_2_.^[^ ^12^, ^22^ ^]^ The combination of the high‐temperature instability of CsPb_2_Br_5_ and volatile nature of PbBr_2_ can promote the reaction CsPb_2_Br_5_ → CsPbBr_3_ + PbBr_2_.^[^ ^8^ ^]^ Additionally, the diffraction pattern of n‐CsPbBr_3_ is similar to the simulated powder diffraction pattern, while the s‐CsPbBr_3_ film shows inhibited growth along the direction [110] and [220], suggesting that the formation of CsPb_2_Br_5_ impurity may disrupt the crystalline regularity of CsPbBr_3_.

The effect of varying CsBr/PbBr_2_ ratios on the morphology and crystal structure of CsPbBr_3_ films was also examined (Figure S1, Supporting Information). Upon increasing the PbBr_2_ ratio (CsBr/PbBr_2_ ratio decreasing from 1 to 0.8), the morphology of the CsPbBr_3_ film tends to show a more densified structure with large crystallite sizes after the post‐ripening process. When the CsBr/PbBr_2_ ratio is 0.85, the film exhibits a uniform morphology with grain sizes of tens of microns and still has a pure orthorhombic phase of CsPbBr_3_ after annealing. However, when the CsBr/PbBr_2_ ratio is further reduced to 0.8, impurity residues cover the surface of the film after annealing although it still has a compact structure. Those impurity grains should be the residual PbBr_2_ left on the surface, indicating that a proper CsBr/PbBr_2_ ratio is required to prepare a compact and impurity‐free CsPbBr_3_ film.

The optical properties of the s‐CsPbBr_3_ and n‐CsPbBr_3_ films were examined. Both films show similar transmittance spectra before and after annealing and have a bandgap of 2.34 eV (Figure 1k). The photoluminescence (PL) spectra of both films show redshift after annealing, which can be ascribed to the effect of reabsorption in the perovskite films (Figure 1l). Indeed, in a perovskite film with larger grain sizes, photons emitted in the material bulk can be reabsorbed by the material and reemit at a lower photon energy on their traveling paths before escaping, leading to an overall redshifted emission. The full‐widths at half maximum (FWHMs) of both films reduce to a smaller value, demonstrating that a much purer phase was obtained after annealing. The electronic states of elements for films were investigated by X‐ray photoelectron spectroscopy (XPS). As shown in Figure S2 (Supporting Information), no significant shift was observed for the Pb 4f peaks in the XPS spectra after annealing, suggesting that no Pb─O bonds were formed despite the annealing in the air.

### 2.2 Measurements and Calculations for Understanding the Morphology Changes During Annealing

**Figure 2:**
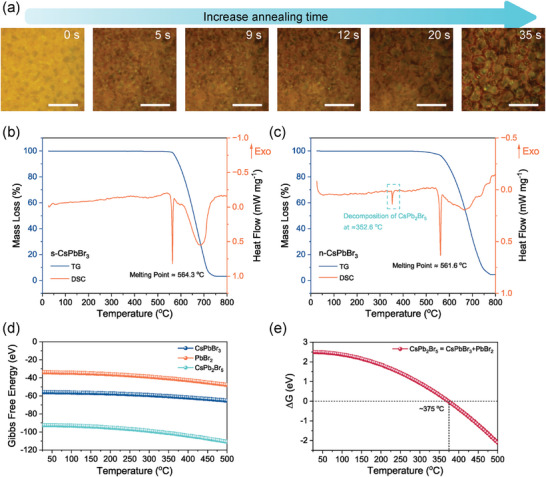
Measurements and calculations for understanding the morphology changes brought about during annealing. a) Snapshots of the in‐situ recorded video showing the morphology revolution of the pristine n‐CsPbBr_3_ film upon annealing. The scale bar represents 40 µm. b,c) TG/DSC analysis of the (b) s‐CsPbBr_3_ and (c) n‐CsPbBr_3_ samples, respectively. The samples were scratched from the pristine films. d,e) Temperature‐dependent Gibbs free energies for (d) CsPb_2_Br_5_, CsPbBr_3_, and PbBr_2_, respectively, and (e) ΔG for the decomposition of CsPb_2_Br_5_.

We further observed the morphology change of the n‐CsPbBr_3_ film during annealing in situ under an optical microscope (Video S1, Supporting Information). The morphology‐changing process was recorded on video and several snapshots are shown in **Figure** **2**a. It reveals that the recrystallization of small grains into larger grains occurs in seconds at 400 °C, after which the grains remain unchanged. There is almost no heat flow and mass loss when further annealing the perovskite films for another 30 min, as confirmed by the TG analysis (Figure S3, Supporting Information). It is interesting to ask why the n‐CsPbBr_3_ has such a significant morphology change while the s‐CsPbBr_3_ retains its morphology after annealing. The thermogravimetry (TG) and differential scanning calorimetry (DSC) analysis reveal that there is no obvious mass loss and endothermic/exothermic peak before 560 °C for the s‐CsPbBr_3_ sample (Figure 2b). Then a sharp endothermic peak was observed at 564 °C, which is assigned to the melting point of CsPbBr_3_. Further increasing the temperature will lead to continuous mass loss, which is accompanied by a broad endothermic peak. This is caused by the decomposition of CsPbBr_3_ into CsBr and PbBr_2_, and the easy sublimation of PbBr_2_ at this temperature range.^[^ ^12^ ^]^ In contrast, the n‐CsPbBr_3_ sample exhibits a sharp endothermic peak at a relatively low temperature of 353 °C (Figure 2c), which is caused by the decomposition of CsPb_2_Br_5_ into CsPbBr_3_ and PbBr_2_, and the subsequent sublimation of PbBr_2_. To further understand this process, the Gibbs free energies of CsPb_2_Br_5_, CsPbBr_3,_ and PbBr_2_ as a function of temperature were calculated (Figure 2d,e). The result shows that the decomposition of CsPb_2_Br_5_ into CsPbBr_3_ and PbBr_2_ occurs at ≈375 °C, which is in accordance with the experimental result.^[^ ^22^ ^]^

From the above results, we can conclude that the recrystallization of CsPbBr_3_ grains is subjected to a typical Ostwald ripening process, and the Pb‐rich impurities in CsPbBr_3_ play a critical role in inducing the Ostwald ripening process at a much lower temperature than its melting point (563 °C). Because the decomposition of CsPb_2_Br_5_ into a mixed CsPbBr_3_/PbBr_2_ phase occurs at a relatively low temperature, the volatile nature of PbBr_2_ facilitates its transport throughout the whole porous film to form a mixed CsPbBr_3_/PbBr_2_ film, thereby inducing the melting point depression and Ostwald ripening of CsPbBr_3_ grains accompanied by the escape of PbBr_2_. Once the sublimation of PbBr_2_ completes, the melting and recrystallization of the grains will also cease. In comparison with other post‐treatment techniques that are treated from the surface, this approach allows the recrystallization of grains from the bottom up to the top surface, enabling the crystals to grow continuously with negligible grain boundaries along the vertical direction, as indicated by the cross‐sectional SEM image (Figure 1i). This transformation from a porous structure into a compact film offers multiple solutions to the aforementioned challenges simultaneously: the thick, compact CsPbBr_3_ film can be prepared at a temperature much lower than its melting point; the mediator PbBr_2_ essentially plays the mediate‐and‐go role and can easily retreat from inside the porous film during the annealing even if for a thick film; and the annealing step enhances the contact between the perovskite film and substrate, inhibiting possible delamination.

### 2.3 Charge Transport Property of the Annealed CsPbBr_3_ Film

**Figure 3:**
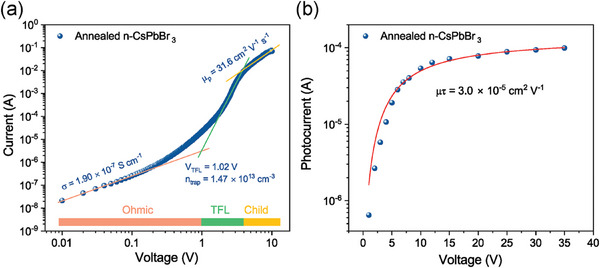
Charge transport property of the annealed n‐CsPbBr_3_ film. a) The space charge‐limited current (SCLC) measurement of the annealed n‐CsPbBr_3_ film with a device structure of FTO/n‐CsPbBr_3_/Carbon. b) Photoconductivity measurement of the annealed n‐CsPbBr_3_ film with a planar device structure making use of two interdigitated gold contacts. A white light‐emitting diode was used as the light source.

The optical and electrical properties of the obtained compact n‐CsPbBr_3_ film were then evaluated. The carrier lifetime of the bare film on glass was obtained from the time‐resolved PL spectra, and the curves were fitted by a biexponential decay [Y = A_1_exp(‐t/τ_1_) + A_2_exp(‐t/τ_2_)], where fast (τ_1_) and slow (τ_2_) components are related to the radiative and nonradiative recombination, respectively.^[^ ^30^, ^31^ ^]^ As displayed in Figure S4 (Supporting Information), after annealing the fast decay lifetime increases from 0.83 to 2.63 ns, and the slow decay lifetime enhances from 5.55 to 13.18 ns, respectively, demonstrating the reduced trap states in the annealed n‐CsPbBr_3_ film. The trap density and carrier mobility in the annealed n‐CsPbBr_3_ film were evaluated by the space‐charge‐limited current (SCLC) measurement with a device structure of FTO/CsPbBr_3_/Carbon (**Figure** **3**a). The trap density (n_trap_) was calculated by the equation: *n_trap_* = 2*V_TFL_* εε_0_/(*eL* ^2^) , and the mobility (µ) was calculated from the Child region by Mott–Gurney's equation: *J_D_* = 9µεε_0_ *V* ^2^/(8*L* ^3^), where V_TFL_ is the trap‐filled limit voltage, L is the film thickness, ε is the relative dielectric constant of CsPbBr_3_ (≈22),^[^ ^32^ ^]^ ε_0_ is the vacuum permittivity, e is the elementary charge, J_D_ is the dark current density. The annealed n‐CsPbBr_3_ film shows a high carrier mobility of 31.6 cm^2^ V^−1^ s^−1^, and this value is very close to that of single crystals,^[^ ^5^, ^33^ ^]^ indicating an excellent charge transport property along the vertical direction. The trap density (1.47 × 10^13^ cm^−3^) is relatively higher than that of single crystals (≈10^10^ cm^−3^)^[^ ^34^ ^]^ but much lower than other reported values in polycrystalline films (≈10^15^ cm^−3^),^[^ ^35^ ^]^ which benefits from the relatively large grain size and low amount of grain boundaries. The mobility‐lifetime (µτ) product was obtained by fitting the curve of photocurrent versus bias voltage dependence using the modified Hecht equation (Figure 3b):^[^ ^4^, ^5^ ^]^

(1) $$I=\frac{I_{0}\mu \tau V}{L^{2}}\;\frac{1-exp\left(-\frac{L^{2}}{\mu \tau V}\right)}{1+\frac{L}{V}\frac{s}{\mu }}$$

where I_0_ is the saturated photocurrent, L is the distance between the electrodes, V is the applied bias, µ is the carrier mobility, τ is the lifetime, and s is the surface recombination rate. The µτ product of the annealed n‐CsPbBr_3_ film was derived to be 3.0 × 10^−5^ cm^2^ V^−1^. This value is lower than that of CsPbBr_3_ single crystal (≈10^−4^ cm^2^ V^−1^),^[^ ^36^ ^]^ which can be due to the relatively higher trap density.

### 2.4 Direct‐Conversion CsPbBr_3_ X‐Ray Detector Device and Its Performance

**Figure 4:**
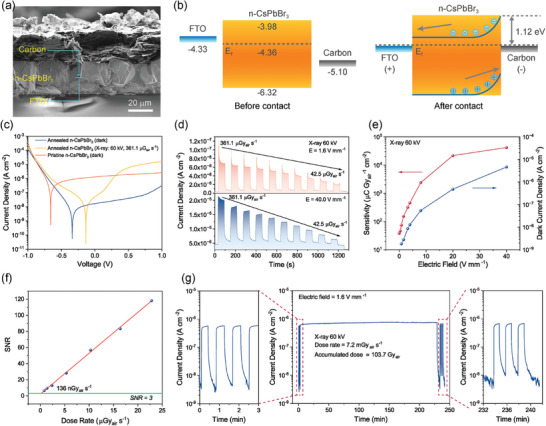
Direct‐conversion CsPbBr_3_ X‐ray detector device and its performance. a) Cross‐sectional SEM image showing the device structure of the CsPbBr_3_ X‐ray detector. b) Schematic energy band diagram for n‐CsPbBr_3_ X‐ray detector before and after contact. c) *J–V* curves measured in the dark and under X‐ray radiation (tube voltage: 60 kV) of the n‐CsPbBr_3_ detector before and after annealing. d) X‐ray response currents of the annealed n‐CsPbBr_3_ detector under applied electric fields of 1.6 and 40 V mm^−1^, respectively. The dose rate of X‐ray radiation is from 361.1 to 42.5 µGy_air_ s^−1^. e) Sensitivity and dark current for the annealed n‐CsPbBr_3_ detector under various applied electric fields. f) SNR of the annealed n‐CsPbBr_3_ detector as a function of X‐ray dose rate. g) Stability of the annealed n‐CsPbBr_3_ X‐ray detector under continuous X‐ray radiation measured at room temperature (28 °C) and 30% relative humidity.

The compact structure accompanied by excellent optoelectronic properties of the annealed n‐CsPbBr_3_ film indicates it can be utilized for highly sensitive X‐ray detection. The X‐ray detector device was then fabricated with a simple structure of FTO/n‐CsPbBr_3_/Carbon (**Figure** **4**a), where the carbon was bladed on top of the CsPbBr_3_ film. The thickness of the annealed n‐CsPbBr_3_ film is ≈25 µm. Figure 4b depicts the energy diagram of the device before and after contact and the work functions of each material were determined either by ultraviolet photoelectron spectroscopy (Figure S5, Supporting Information) or from literature.^[^ ^24^, ^26^ ^]^ The energy diagram reveals that an Ohmic contact and a Schottky junction are formed at the FTO/n‐CsPbBr_3_ interface and the n‐CsPbBr_3_/Carbon interface, respectively. The *J–V* curves of the device show a typical rectifying behavior (Figure 4c), verifying the formation of the Schottky junction. The dislocation of the current minimum from 0 V is due to the ion migration‐induced hysteresis, which can be commonly observed in perovskite‐based devices.^[^ ^37^, ^38^, ^39^, ^40^ ^]^ Furthermore, the annealed n‐CsPbBr_3_‐based device exhibits a much lower dark leakage current density on the reverse side, at only 4.00 × 10^−8^ A cm^−2^ under a bias of 0.5 V, than that of the pristine n‐CsPbBr_3_‐based device, which is 1.95 × 10^−6^ A cm^−2^ under the same bias. Such a low reverse leakage current arises from a more compact structure and lower defect states of the annealed film, as expected from the TRPL analysis. Under X‐ray radiation, the photocurrent of the annealed n‐CsPbBr_3_‐based detector increases intensively with the bias, especially when the bias surpasses 0.1 V, which is expected to yield a high sensitivity. The dark *J–V* curves of CsPbBr_3_ detectors with various CsBr/PbBr_2_ ratios in the precursor solution were also examined (Figure S6, Supporting Information). It reveals that the device with a ratio of 0.92 has the lowest dark current at the reverse bias, which is much lower than the s‐CsPbBr_3_‐based device. Further decreasing the CsBr/PbBr_2_ ratio to below 0.85 will increase the dark current and the corresponding *J–V* curves show a symmetric characteristic, suggesting the formation of Ohmic contacts on both sides. This is, presumably, due to the various CsBr/PbBr_2_ ratios in the precursor that can result in different defect types and densities in the CsPbBr_3_ films, thus changing their Fermi level and electrical properties.^[^ ^41^ ^]^ While this is an interesting observation, because it is not the focus of this work and the related device shows a much higher dark current, we do not explore this feature further. Therefore, the optimized CsBr/PbBr_2_ ratio in the precursor was chosen to be 0.92 in terms of the dark current unless otherwise specified.

The current density profiles of the detector in dependence on dose rate were recorded at 1.6 and 40 V mm^−1^, respectively, as shown in Figure 4d. Under the electric field of 1.6 V mm^−1^, the detector shows a spike when the X‐ray radiation is suddenly on regardless of the X‐ray dose rate. This current spike arises from the inefficient charge collection by the electrode under a low electric field. By contrast, under the electric field of 40 V mm^−1^, the spike almost disappears regardless of the dose rate, suggesting a very fast collection of photo carriers. The bias dependence of sensitivity was measured (Figure 4e). Under the low electric field of 1.6 V mm^−1^, the device shows an extremely low steady‐state dark current density of 2.8 × 10^−9^ A cm^−2^, but the sensitivity also exhibits a relatively low value of 153.0 µC Gy_air_^−1^ cm^−2^. With increasing the applied electric field, both the sensitivity and dark current of the device increase. Under a high electric field of 40 V mm^−1^, the steady‐state dark current reaches 4.6 × 10^−6^ A cm^−2^, and the sensitivity increases by more than two orders of magnitude to 4.2 × 10^4^ µC Gy_air_^−1^ cm^−2^. Such an intensive increase of sensitivity at high bias arises from the charge injection from electrodes due to the reduced energy barrier at the interface, as demonstrated in our previous report.^[^ ^26^ ^]^ It should be noted that the thickness of the perovskite is only 25 µm, but the sensitivity is comparable to or even higher than those of the reported devices with larger perovskite layer thicknesses operated under a similar electric field (Table S1, Supporting Information).^[^ ^10^, ^41^, ^42^ ^]^ The sensitivity can be further increased at a higher electric field, but it will inevitably give rise to a higher dark current. The photoconductive gain, G, of the device under various electric fields and dose rates was estimated and the calculation details are given in Supporting Information, Note S1. As shown in Figure S7 (Supporting Information), as the electric field increases from 0.4 to 40 V mm^−1^, the gain factor increases by more than two orders of magnitude, from 5.8 × 10^2^ to 3.8 × 10⁵. This behavior is consistent with the significantly enhanced charge injection from the electrode at the higher electric fields. Notably, at the low electric fields, the gain factor decreases with increasing dose rate due to the relatively low charge extraction efficiency, whereas the high electric fields, e.g., 40 V mm^−1^, promote the charge extraction and allow the gain factor to stabilize across various dose rates. The response sensitivity of the device to X‐rays with various energies was also measured. As shown in Figures S8 and S9 (Supporting Information), the sensitivity decreases with increasing the energy of X‐rays, which may be caused by the relatively low perovskite thickness and thus the insufficient absorption of X‐ray photons. Nevertheless, the sensitivity is still above 10^4^ µC G_yair_^−1^ cm^−2^ at the 120 kV X‐ray radiation.

The limit of detection (LoD) was evaluated by calculating the signal‐to‐noise (SNR) as a function of dose rate and defined as the dose rate when the detector yielded an SNR of 3. As given in Figure S10 (Supporting Information), with the reduction of the X‐ray dose rate, the response signal becomes weaker. The current induced by 60 kV X‐rays with a dose rate of 778 nGy_air_ s^−1^ is still distinguishable from the noise and shows an SNR of 6.7. By fitting the SNR versus dose rate (Figure 4f), the LoD is identified as low as 136 nGy_air_ s^−1^. Such a low detection limit is due to the low dark current under a low electric field and suggests a good detection of weak X‐rays. The operation stability of the n‐CsPbBr_3_ X‐ray detector was evaluated by tracing the photocurrent with 60 kV X‐ray at a high dose rate of 7.22 mGy_air_ s^−1^ for ≈240 min (Figure 4g). The detector only shows slight degradation under an accumulated X‐ray dose of 104 Gy_air,_ which is equivalent to half a million posteroanterior chest examinations in consideration of the entrance surface dose of 0.2 mGy_air_ for chest radiography. There is barely any dark current drift during and after the measurement, indicating negligible ion migration in the film under the measurement condition. In contrast, the s‐CsPbBr_3_ X‐ray detector is much less stable alongside large fluctuations in photocurrent (Figure S11, Supporting Information), which may result from the higher defect density and smaller grain size of the corresponding perovskite film. We further evaluated the stability of the n‐CsPbBr_3_ X‐ray detector at various operating temperatures and humidities. As shown in Figure S12 (Supporting Information), the device demonstrates high stability at room temperature with a relative humidity of 50%. Also, the device maintains its stability at temperatures up to 60 °C, while it exhibits significant fluctuations and degradations in photocurrent at 90 °C, likely due to severe ion migration arising at the elevated temperature.

### 2.5 Real‐Time Dynamic X‐Ray Imaging using Flat‐Panel Detector

**Figure 5:**
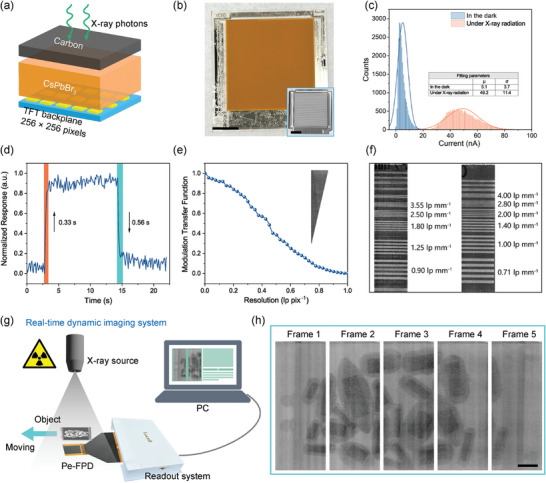
Flat‐panel X‐ray imaging performance. a) Schematic of CsPbBr_3_ perovskite film integration with TFT backplane. b) CsPbBr_3_ film deposited on a LinkZill TFT backplane (Model: YDS5). The inset image on the bottom right shows the bare TFT backplane. Scale bar: 1 cm. c) The dark and light‐field gray scale distribution of the X‐ray FPD. d) Transient response of the X‐ray FPD. e) MTF curve of the X‐ray FPD. The inset is the X‐ray image of tungsten plate. f) X‐ray images of a standard resolution pattern in different regions. g) Schematic diagram of the real‐time dynamic imaging system. h) Several frames from the real‐time dynamic X‐ray imaging video at various times. Scale bar: 0.5 cm.

The capability of the n‐CsPbBr_3_ X‐ray detector for direct imaging was evaluated. We first imaged a chicken drumette using the device with a single pixel size of 1 mm by 2 mm at various x‐ray dose rates. As shown in Figure S13 (Supporting Information), a very clear image was achieved under a dose rate of 183 µGy_air_ s^−1^, and from the image the bone and tissue can be well distinguished. The contrast of the image decreased as the dose rate decreased. When the dose rate was reduced to 7.1 µGy_air_ s^−1^, one can only recognize the shape of the drumette. Subsequently, we integrated the all‐inorganic n‐CsPbBr_3_ film with a commercial indium─gallium─zinc─oxide (IGZO) thin‐film transistor (TFT) array (**Figure** **5**a). The dimension of the TFT array is 44.6 × 46.6 mm^2^ with an active area of 32 × 32 mm^2^. The pixel size is 126 × 126 µm^2^ and the resolution is 256 × 256. The n‐CsPbBr_3_ film was deposited on the TFT array by the same method as on the FTO substrate. To avoid destroying the TFT array, we applied a very gentle annealing process on the pristine n‐CsPbBr_3_ film. As shown in Figure 5b, the perovskite film is very uniform across the entire backplane. After blading the carbon electrode and bonding with a flexible printed circuit (FPC), the perovskite flat panel detector (Pe‐FPD) was connected to the readout system for X‐ray detection measurement (Figure S14, Supporting Information). The pixel's gray‐scale values of the Pe‐FPD were fitted by normal distribution curves to derive the dead pixel rate (Figure 5c), as described in the experimental section. In the dark, the gray‐scale values in the dark show a narrow distribution with a standard deviation of 3.7. Under X‐ray exposure, the gray‐scale values show a wider distribution with a standard deviation of 11.4, which can be partially due to the point illumination of the X‐ray source. From the current values observed under X‐ray exposure, an extremely low dead pixel rate of 0.05% was determined. The Pe‐FPD also displays a rapid rise time and fall time at 0.33 s and 0.56 s, respectively (Figure 5d). Note that these response speeds might be underestimated because of the limited readout speed of the readout system at 9 frame‐per‐second (fps). The spatial resolution of the Pe‐FPD was determined by the modulation transfer function (MTF) using the slanted‐edge method (Figure 5e)^[^ ^43^ ^]^ The spatial resolution of the compact n‐CsPbBr_3_‐based FPD achieves 0.65 lp pix^−1^ at MTF = 0.2, which is equivalent to 5.16 lp mm^−1^ considering a pixel size of 126 µm. This value is among the highest values for the reported Pe‐FPDs, as summarized in Table S2 (Supporting Information). Also, in comparison with commercial X‐ray detectors (Table S3, Supporting Information), the high sensitivity and low cost of our perovskite X‐ray detector suggest its high potential for future commercial applications. Digital radiography (DR) images (Figure 5f) of a standard line pair pattern (Figure S15, Supporting Information, Model: Type 18 D, QUART GmbH, Germany) were obtained, from which 4.00 lp mm^−1^ can be well resolved. One might wonder why this value is lower than the one obtained from the MTF measurement (5.16 lp mm^−1^). This discrepancy could arise from the finite distance between the standard pattern card and the Pe‐FPD. More specifically, the standard pattern card is made of thin lead (0.1 mm) encased in a plastic having a thickness of ≈1 mm, giving rise to a 1 mm space between the pattern card and the Pe‐FPD. However, for the MTF measurement, a thin tungsten plate (1 mm) was placed directly on the Pe‐FPD, leaving almost no space between the plate and the Pe‐FPD. Given that the X‐ray source is a point source, the greater distance between the standard pattern card and the Pe‐FPD can cause image blurring, and thus only 4 lp mm^−1^ could be distinguished in this case. This was also confirmed in an experiment that measured the MTF by placing a 1 mm plastic sheet between the tungsten plate and the Pe‐FPD and obtained a resolution of 3.5 lp mm^−1^ (Figure S16, Supporting Information).

Finally, real‐time dynamic imaging was achieved with our Pe‐FPD (Figure 5g), as displayed in Video S2 (Supporting Information). The video was recorded at a readout speed of 9 fps by moving the magnetic polytetrafluoroethylene (PTFE) stir bars (Figure S17, Supporting Information) under continuous X‐ray exposure. Figure 5h presents several frames from the video at various times, which display a high resolution and contrast. From the video, the PTFE shell and magnet core can be clearly recognized and no motion artifacts are observed, suggesting the excellent imaging property of our Pe‐FPD and its high potential in fast X‐ray imaging applications.

## 3 Conclusion

In this study, we establish a nonstoichiometry‐enabled strategy for producing thick, dense CsPbBr_3_ perovskite films. Through annealing treatment, the surplus PbBr_2_ interacts with the CsPbBr_3_ bulk grains, leading to a depression in the melting point and the subsequent under‐temperature Ostwald ripening of the CsPbBr_3_ crystalline domains. This process transforms the porous CsPbBr_3_ film with small grains into a compact film characterized by significantly enlarged grains synchronized with the removal of the volatile PbBr_2_, all achieved at temperatures much lower than the perovskite's melting point. The resulting compact CsPbBr_3_ film exhibits exceptional optoelectronic properties. Utilizing this approach, we developed a highly sensitive all‐inorganic X‐ray detector, boasting a sensitivity of 4.2 × 10^4^ µC Gy_air_^−1^ cm^−2^ and a low detection limit of 136 nGy_air_ s^−1^, coupled with outstanding operational stability. Additionally, the X‐ray flat‐panel detector based on the compact film achieved an impressive resolution of 0.65 lp pix^−1^ at MTF = 0.2, facilitating real‐time dynamic imaging for the first time. We anticipate that this methodology holds great promise for fabricating large‐scale, highly stable, and sensitive Pe‐FPDs suitable for applications in medical imaging and security screening.

## 4 Experimental Section

### Preparation of Perovskite Film and X‐Ray Detector

To prepare the perovskite precursor solution, PbBr_2_ (734 mg) and CsBr (426, 417, 392, 362, and 340 mg, respectively) were added into a mixed DMF/DMSO (45mL/5mL) solvent with a concentration of 0.04 m, which was then stirred at room temperature for 3 h in ambient condition. The CsBr/PbBr_2_ ratios were adjusted to be 1, 0.92, 0.98, 0.85 and 0.8, respectively. Then the solution was transferred into a glass tube, which was deposited on an FTO substrate to form a perovskite film by an aerosol–liquid–solid (ALS) method, as reported previously.^[^ ^21^, ^23^, ^24^, ^25^, ^26^ ^]^ Briefly, the perovskite solution was ultrasonically atomized to form an aerosol and carried by nitrogen gas to be deposited on FTO substrates at 200 °C. The nitrogen gas flow rate was set to be 1.5 L min^−1^ and the distance between the nozzle and substrate was 2 mm. The thickness of the perovskite films was adjusted by controlling the number of depositions. After deposition, the perovskite films were transferred on a 400 °C hot plate for 5 min and then cooled down to room temperature at a slow rate. All the deposition procedures were conducted in ambient conditions. To fabricate the X‐ray detector device, a carbon electrode was bladed onto the perovskite layer using a mask followed by annealing at 120 °C for another 15 min and slowly cooling down. The active area of the device is 0.02 cm^2^.

### Perovskite Film Characterization

SEM images were obtained on a field‐emission scanning electron microscopy (JEOL 7800F). XRD patterns were collected on Rigaku Smartlab using Cu Kα radiation (λ = 1.5418 Å). TG/DSC was conducted on a simultaneous thermal analyzer (Netzsch, STA449F3) from 25 to 800 °C in the air with a temperature ramping rate of 5 °C min^−1^. Optical transmittance and steady‐state photoluminescence (405 nm excitation) spectra were obtained on QSpec LTF‐3000 (Biaoqi Optoelectronics, China). TRPL was conducted on a time‐resolved, single‐photon counting instrument (pico 1000, Dalian Institute of Chemical Physics, China). XPS spectra were performed on Shimadzu/Kratos AXIS SUPRA+. UPS spectra were carried out on ESCALAB Xi+ (Thermo Fisher Scientific).

### X‐Ray Detection Characterization

The X‐ray detection experiments were performed in a closed lead box equipped with a medical X‐ray tube (HPX‐160‐11, Varex Imaging) with a maximum peak voltage of 160 kV. The target material is tungsten (W) with an angle of 11^°^ and the assembly permanent filtration is 0.8 mm Be. All the X‐ray response characterization was conducted in air in the dark. The accelerating voltage was from 30 to 120 kV, and the current was from 0.1 to 1 mA to change the x‐ray dose rate. Unless otherwise noted, the voltage for X‐rays refers to the tube voltage. For measuring the limit of detection, Al foils with various thicknesses were inserted between the X‐ray source and sample device as the attenuator. The radiation dose rate was carefully calibrated with a dosimeter (AT1123, ATOMTEX). The *I–t* and *I–V* curves in the dark and under radiation were carried out with a Keithley 2612B source meter. The sensitivity was calculated by using the net photocurrent induced by the X‐ray photons divided by dose rate. The SNR was calculated by:^[^ ^26^ ^]^

(2) $$SNR=J_{s}/J_{n}$$

where J_s_ is the net X‐ray generated current density and J_n_ is the standard deviation of the photocurrent density under X‐ray exposure.

The TFT backplane (Model: YDS5) and the readout system (Model: TruEbox 03MR (256 × 256)) are provided by LinkZill Technology Co. Ltd. The backplane consists of an indium─gallium─zinc─oxide (IGZO) TFT array, whose dimension is 44.6 × 46.6 mm^2^ with an active area of 32 × 32 mm^2^. The pixel size is 126 × 126 µm^2^ and the resolution is 256 × 256. The readout speed is 9 frames per second. The current distribution in the dark and light fields of the Pe‐FPD was fitted to a normal distribution curve, as reported elsewhere:^[^ ^44^ ^]^

(3) $$f\;\left(x\right)=\frac{1}{\sqrt{2\pi }\sigma }\;exp\left(-\frac{{\left(x-\mu \right)}^{2}}{2\sigma^{2}}\right)$$

where µ is the average current value and σ is the standard deviation. Dead pixel rate is defined as the rate of those whose current values fall outside the range µ ± 4σ in all pixels. The MTF was determined by the slanted‐edge method. A thin tungsten plate was placed on the X‐ray detector and the MTF was derived from the obtained X‐ray image. The real‐time dynamic imaging was obtained by moving the objects between the X‐ray source and the FPD.

### Computational Methods

The density functional theory (DFT) calculations were carried out with the VASP code.^[^ ^45^ ^]^ The Perdew–Burke–Ernzerhof (PBE) functional within generalized gradient approximation (GGA) was used to process the exchange–correlation,^[^ ^46^ ^]^ while the projectoraugmented‐wave pseudopotential (PAW) was applied with a kinetic energy cut‐off of 500 eV,^[^ ^47^ ^]^ which was utilized to describe the expansion of the electronic eigenfunctions. The Brillouin‐zone integration was sampled by a Γ‐centered 5 × 5 × 5 Monkhorst–Pack k‐point. All atomic positions were fully relaxed until energy and force reached a tolerance of 1 × 10^−5^ eV and 0.03 eV Å^−1^, respectively. The dispersion‐corrected DFT‐D method was employed to consider the long‐range interaction.^[^ ^48^ ^]^ The Gibbs free energy change (ΔG) was calculated by computational hydrogen electrode (CHE) model as follows:

(4) ΔG=ΔE+ΔZPE−TΔS

where ΔE is the reaction energy obtained by the total energy difference between the reactant and product molecules absorbed on the catalyst surface and ΔS is the change in entropy for each reaction, ΔZPE is the zero‐point energy correction to the Gibbs free energy. T represents room temperature.

## Conflict of Interest

The authors declare no conflict of interest.

## Author Contributions

J.W. and S.S.Y. contributed equally to this work. J.W. and S.H.Y. conceived the idea. J.W. and S.S.Y. conducted the fabrication and characterization of the devices. H.J. and Y.L. helped with the first‐principle calculation and the X‐ray imaging. K.Z. assisted with the characterizations of materials. D.L.P revised the manuscript. All authors have approved the manuscript.

## Supporting information



Supporting Information

Supplemental Video 1

Supplemental Video 2

## Data Availability

The data that support the findings of this study are available from the corresponding author upon reasonable request.
