# Supplementary material for: Nonstoichiometry Promoted Solventless Recrystallization of a Thick and Compact CsPbBr3 Film for Real‐Time Dynamic X‐Ray Imaging
Source: Adv Sci (Weinh). 2024 Oct 21;11(46):2407314. doi: 10.1002/advs.202407314 (PMC11633472; doi:10.1002/advs.202407314)
Supplement: Supplementary file 1 — Supporting Information [file ADVS-11-2407314-s002.docx]

Supporting Information

**Nonstoichiometry promoted solventless recrystallization of a thick and compact CsPbBr_3_ film for real-time dynamic X-ray imaging**

*Jian Wang,^*^ Shanshan Yu, Handong Jin, Yu Li, Kai Zhang, David Lee Phillips, Shihe Yang^*^*

Dr. J. Wang, S. Yu, Y. Li, H. Jin, K. Zhang, Prof. S. Yang

Institute of Biomedical Engineering, Shenzhen Bay Laboratory, Shenzhen, Guangdong 518107, China

E-mail: jianwsolar@pku.edu.cn; chsyang@pku.edu.cn

Dr. J. Wang, S. Yu, Y. Li, H. Jin, K. Zhang, Prof. S. Yang

Guangdong Provincial Key Lab of Nano-Micro Materials Research, School of Advanced Materials, Shenzhen Graduate School, Peking University, Shenzhen, Guangdong 518055, China.

S. Yu, Prof. D. L. Phillips

Department of Chemistry, The University of Hong Kong, Hong Kong S.A.R., China


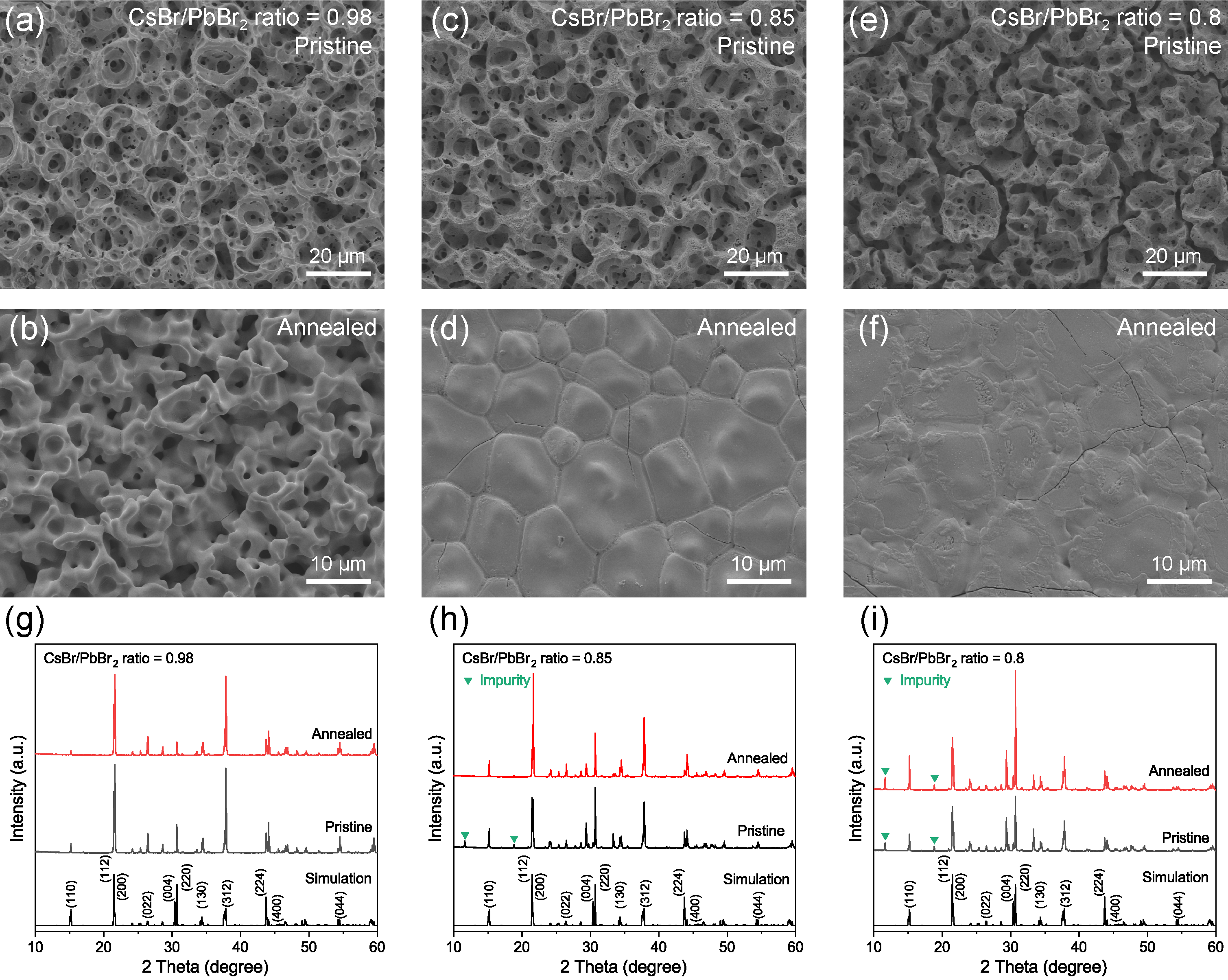


**Figure S1.** Characterization of n-CsPbBr_3_ films with various CsBr/PbBr_2_ ratios. (a-f) SEM images of CsPbBr_3_ films prepared with CsBr/PbBr_2_ ratios of 0.98 (a and b), 0.85 (c and d), and 0.8 (e and f) before (a, c and e) and after (b, d and f) annealing. (g-i) XRD patterns of CsPbBr_3_ films prepared with CsBr/PbBr_2_ ratios of 0.98 (g), 0.85 (h) and 0.8 (i) before and after annealing.


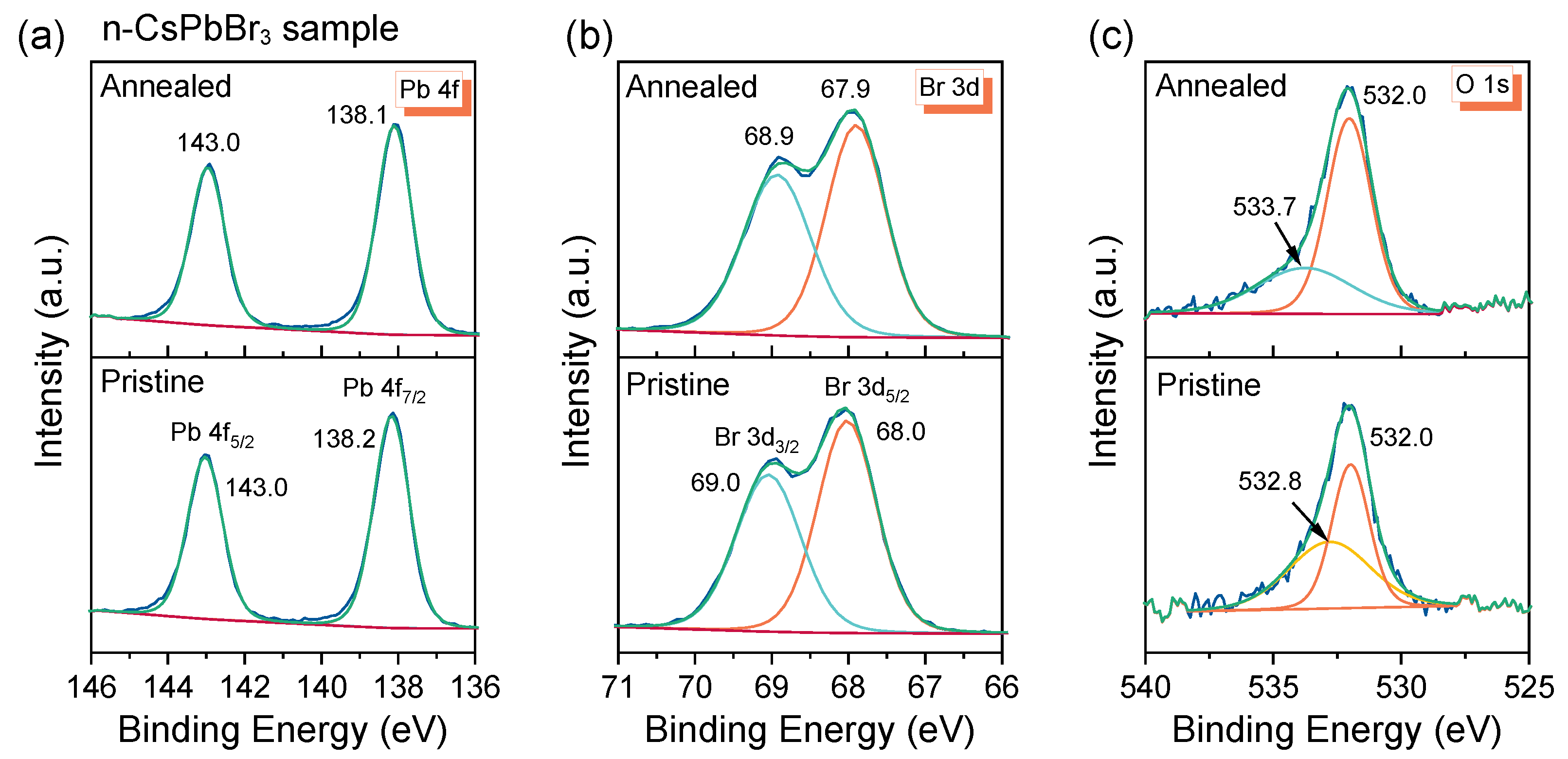


**Figure S2.** XPS spectra of the n-CsPbBr_3_ sample before and after annealing.


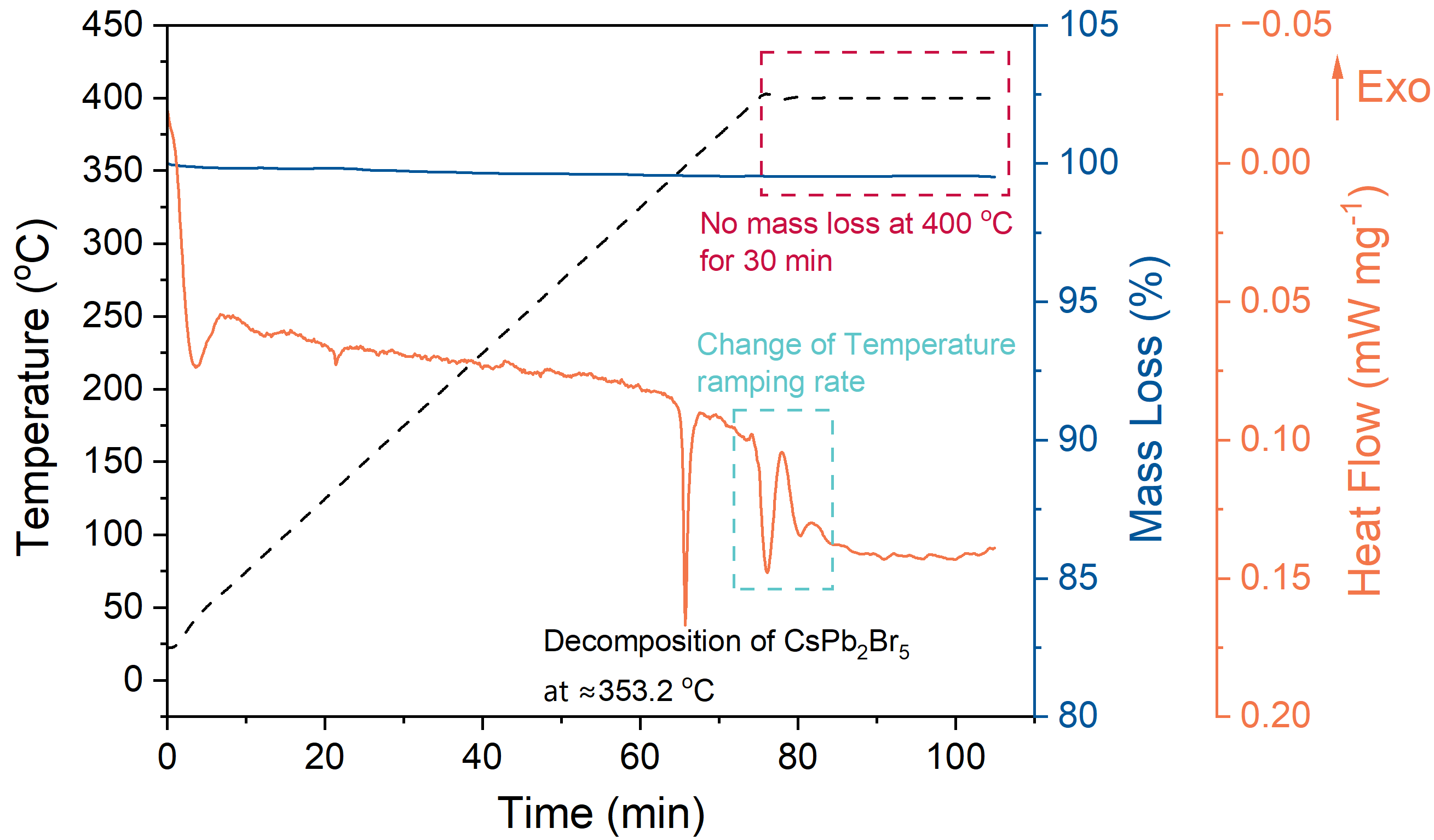


**Figure S3.** TG/DSC analysis of the n-CsPbBr_3_ sample with annealing at 400 ^o^C for 30 min. The sample was scratched from the pristine deposited film.


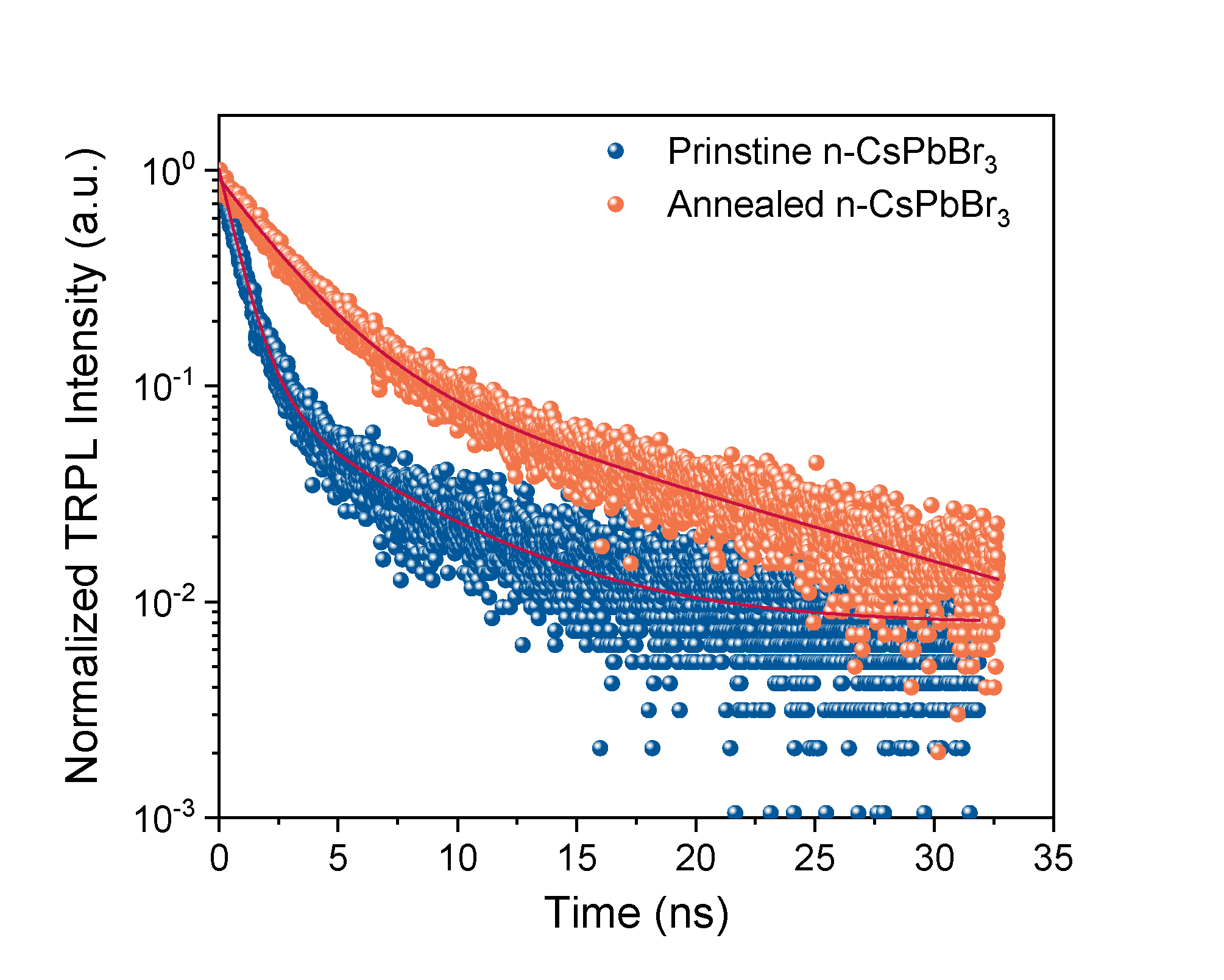


**Figure S4.** Normalized time-resolved photoluminescence (TRPL) decay of the pristine and the annealed n-CsPbBr_3_ films.


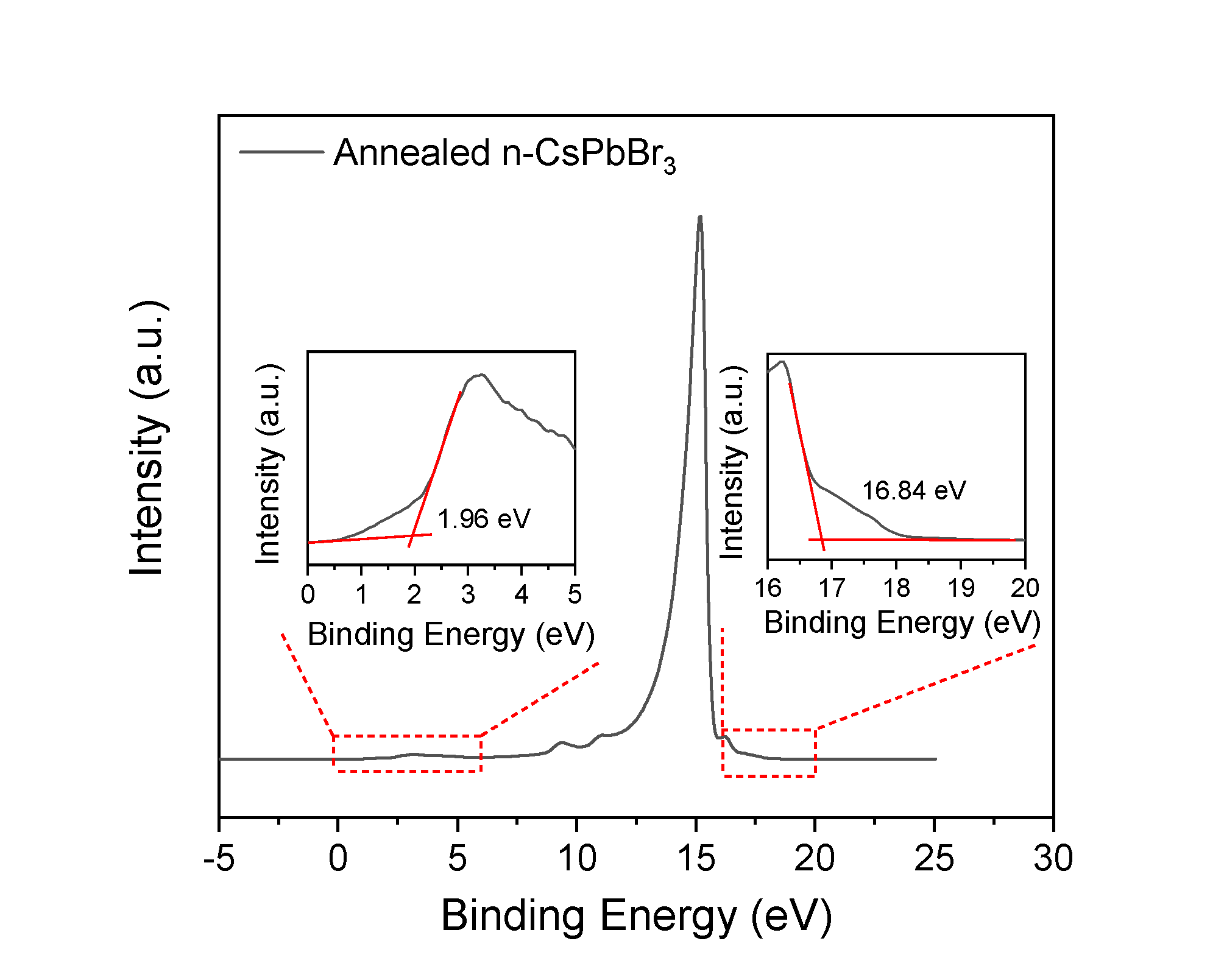


**Figure S5.** Ultraviolet photoelectron spectroscopy (UPS) measurement of the annealed n-CsPbBr_3_ film.


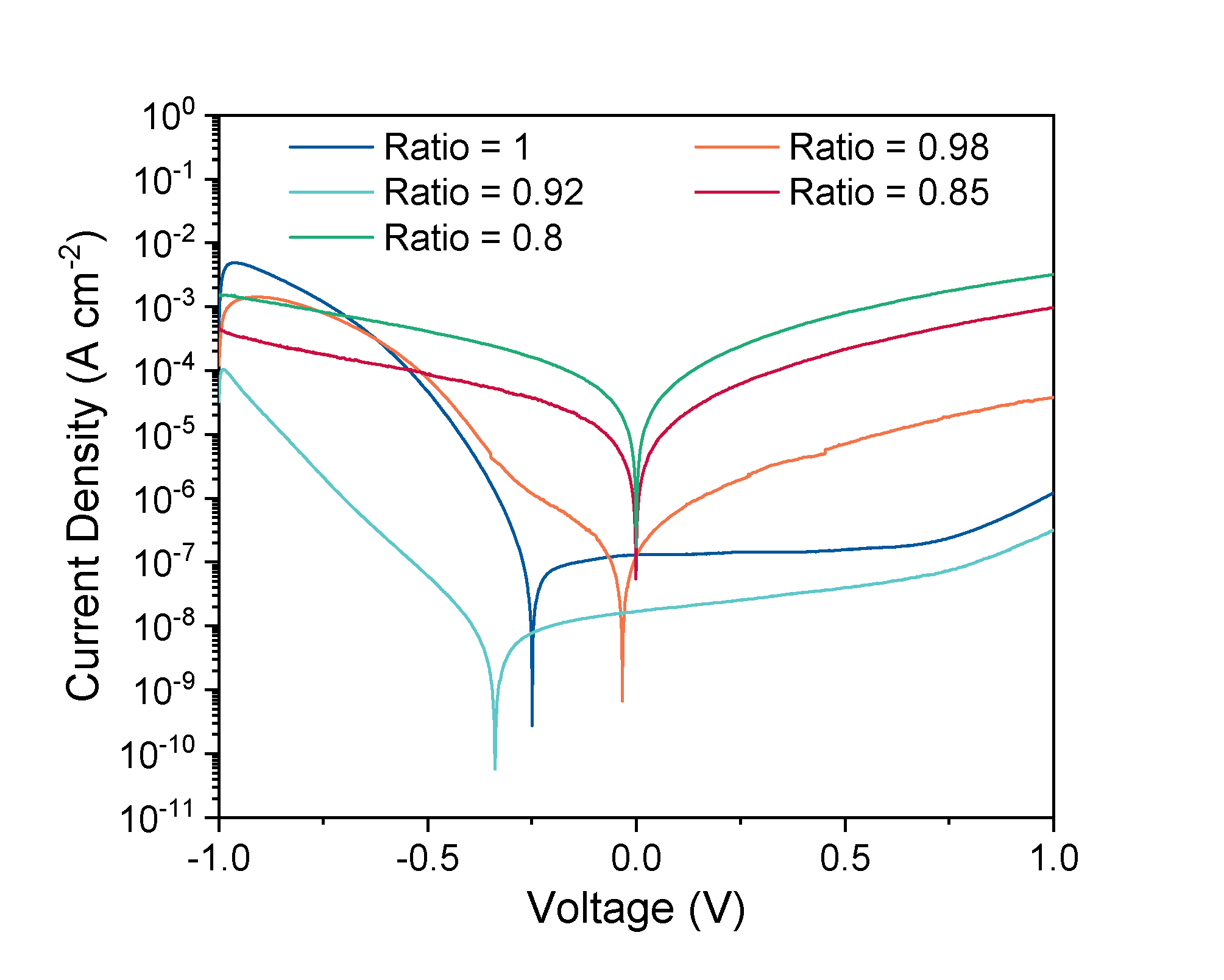


**Figure S6.** J-V curves measured in the dark for the CsPbBr_3_ X-ray detector with various CsBr/PbBr_2_ ratios in the precursor.


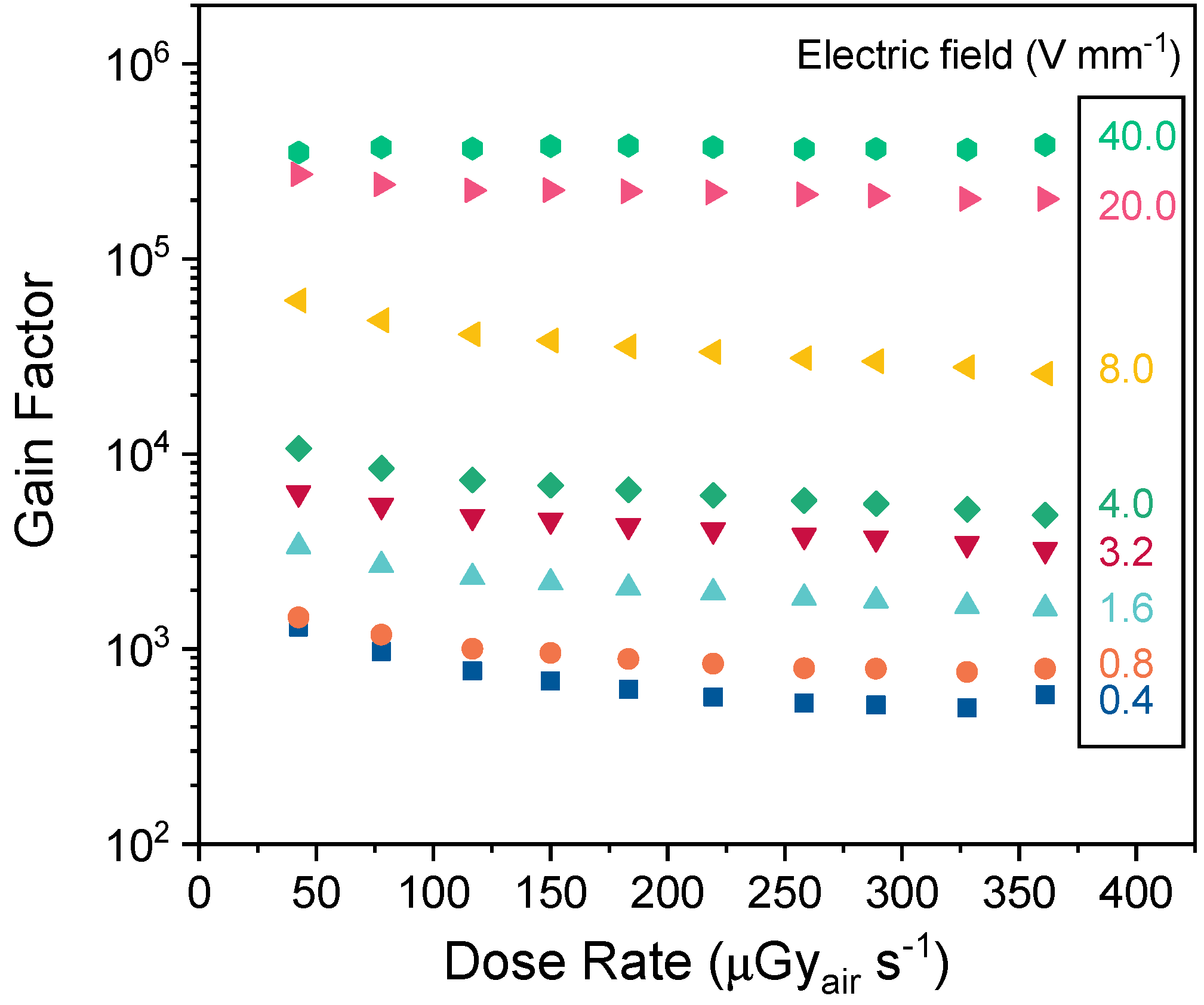


**Figure S7.** Photoconductive gain of the device at various electric fields and dose rates.

**Note S1:** Calculation of photoconductive gain

The photoconductive gain, G, of the device under various electric fields and dose rates was estimated by the following equations:^[1, 2]^

$J_{t}={\phi\beta e}/A$ (1)

$\phi={\varepsilon Dm_{s}}/{E_{ph}}$ (2)

$\varepsilon=1-exp\left( -\alpha L \right)$ (3)

$\beta={E_{ph}}/{W_{\pm}}$ (4)

$W_{\pm}=2E_{g}+1.43$ (5)

$G={J_{p}}/{J_{t}}$ (6)

where *J_t_* is the theoretical current induced by X-ray irradiation, *J_p_* is the net photocurrent obtained by the device, *φ* is the photon absorption rate, *β* is the maximum number of carriers excited by an X-ray photon, *e* is the elementary charge, *A* is the active area, *ε* is the attenuation ratio, *D* is the dose rate, *m_s_* is the sample mass, *E_ph_* is the photon energy (60 keV), *α* is the attenuation coefficient, *W_±_* is the ionization energy, and *E_g_* is the bandgap of perovskite.


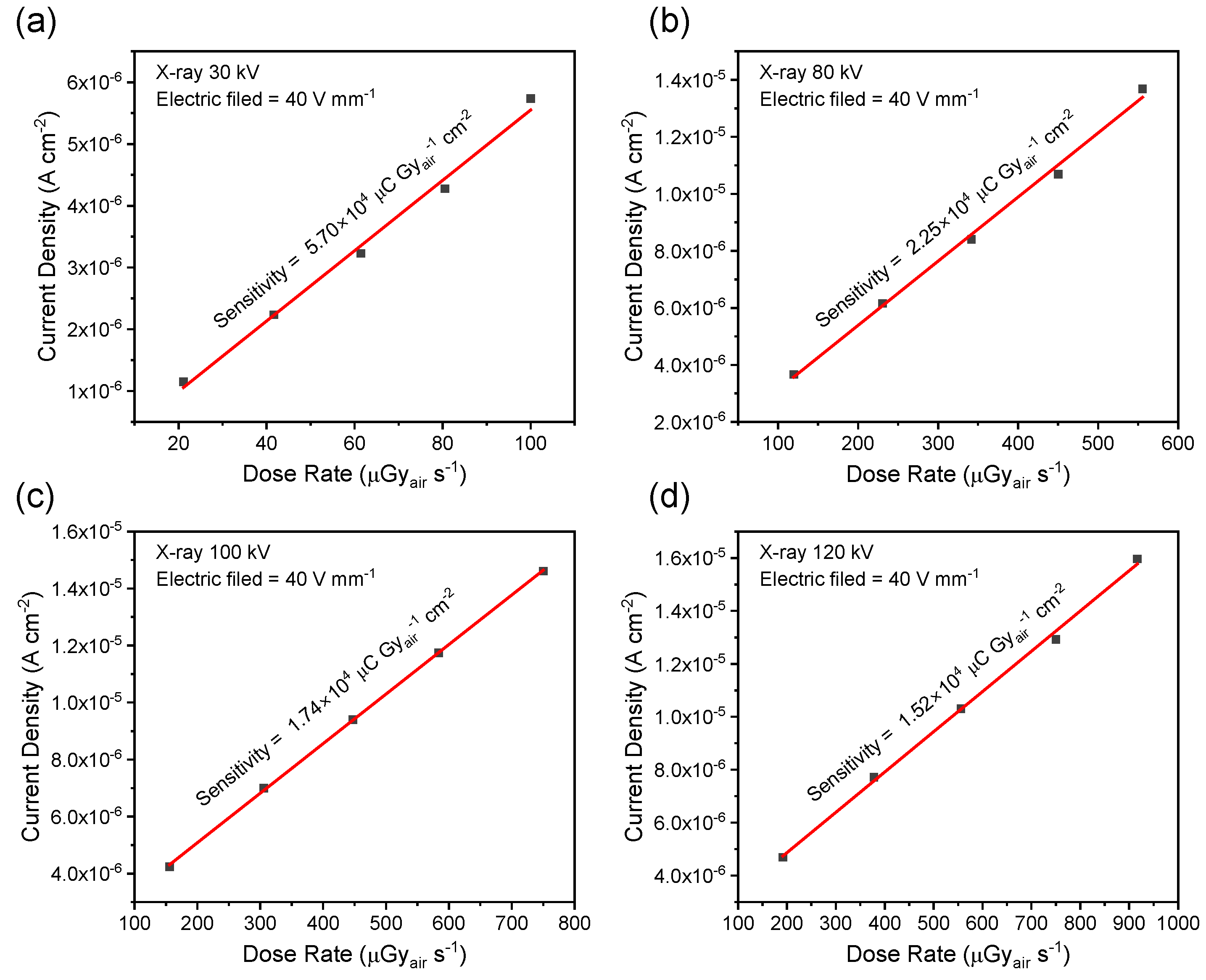


**Figure S8.** Sensitivity of the n-CsPbBr_3_ X-ray detector under X-ray irradiation with various photon energies: 30 kV (A), 80 kV (B), 100kV (C) and 120 kV (D). The CsBr/PbBr_2_ ratio in the precursor is 0.92.


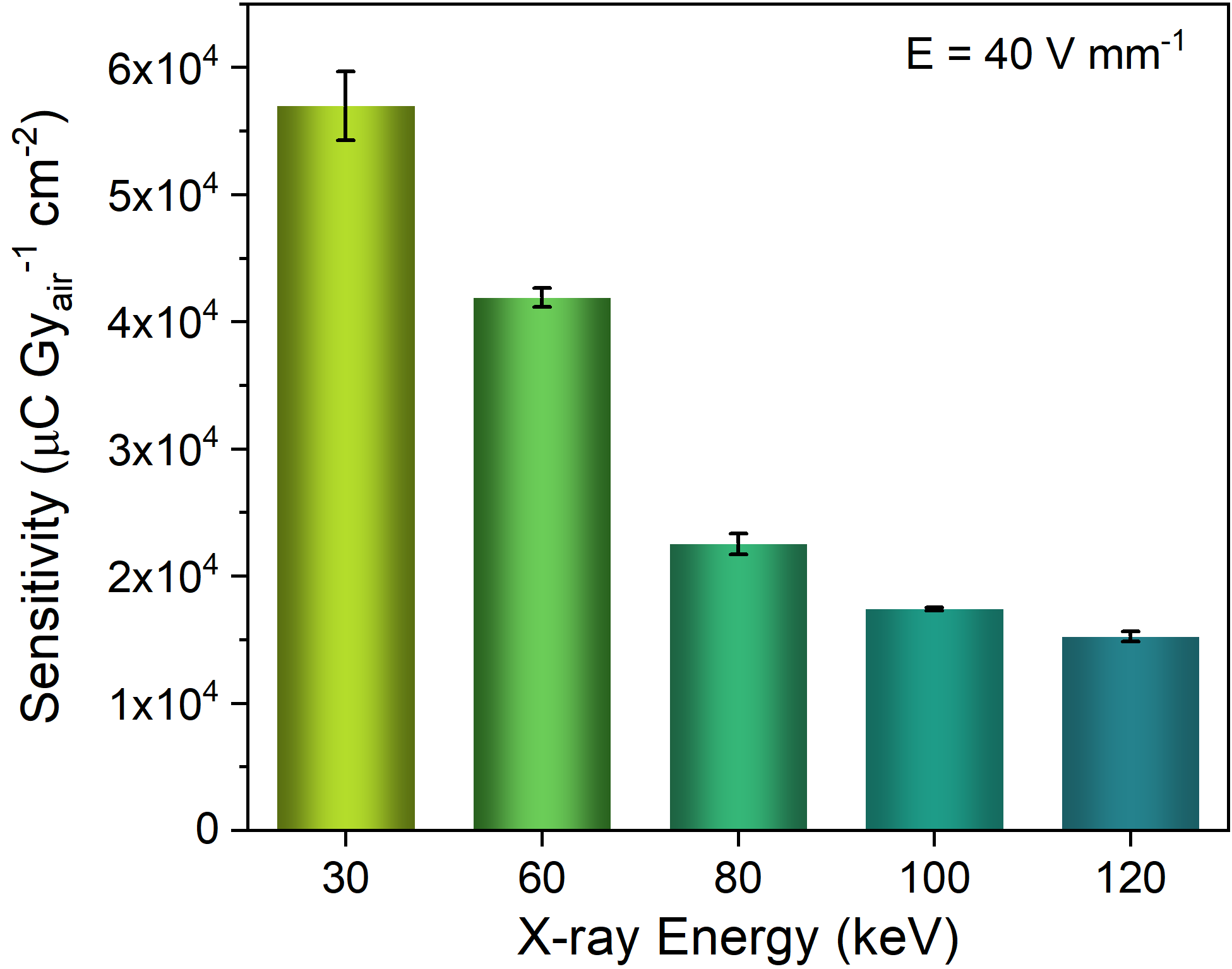


**Figure S9.** Sensitivity comparison for the CsPbBr_3_ X-ray detector under X-rays with various photon energies.


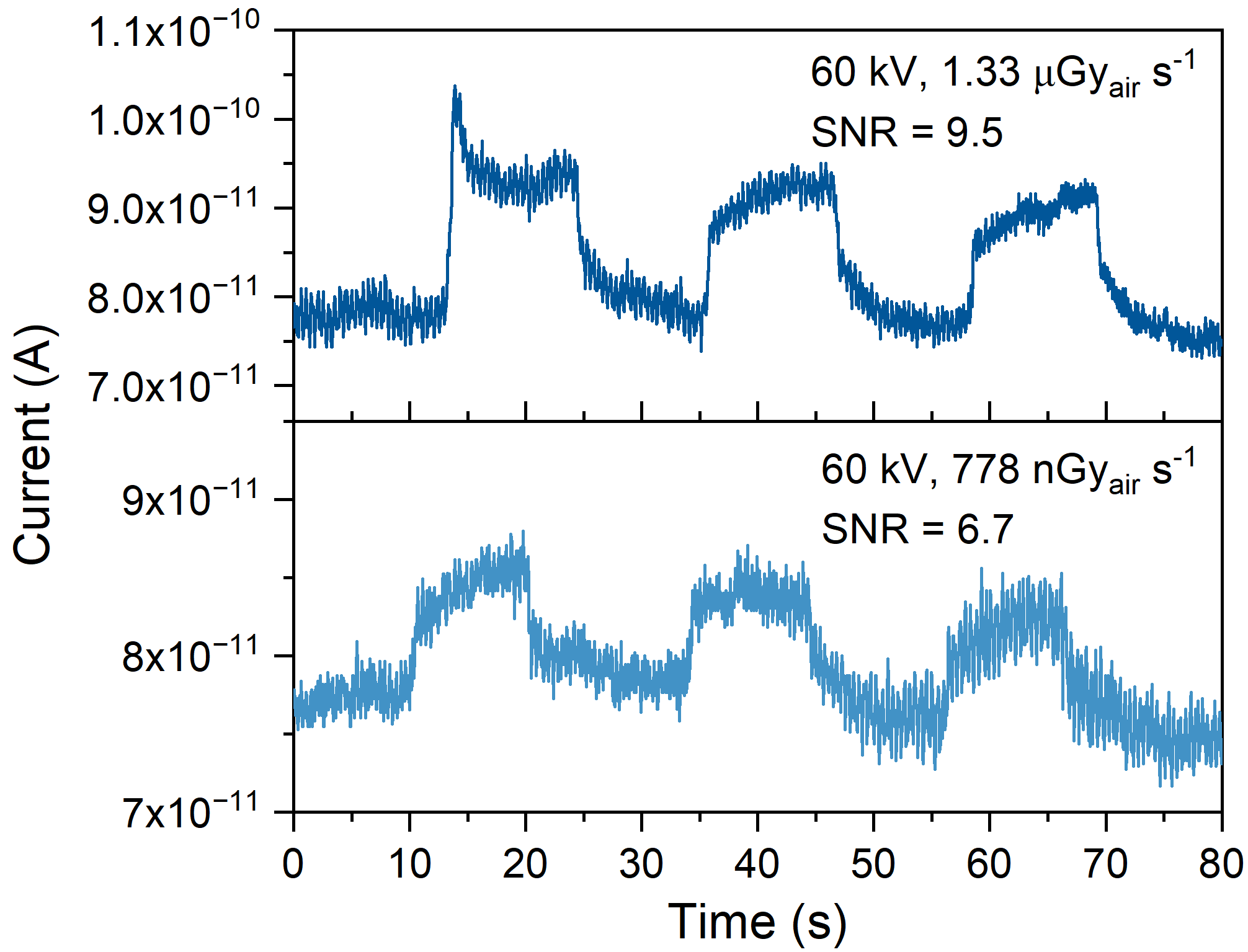


**Figure S10.** X-ray response current of the n-CsPbBr_3_ X-ray detector under low-dose X-ray irradiation.


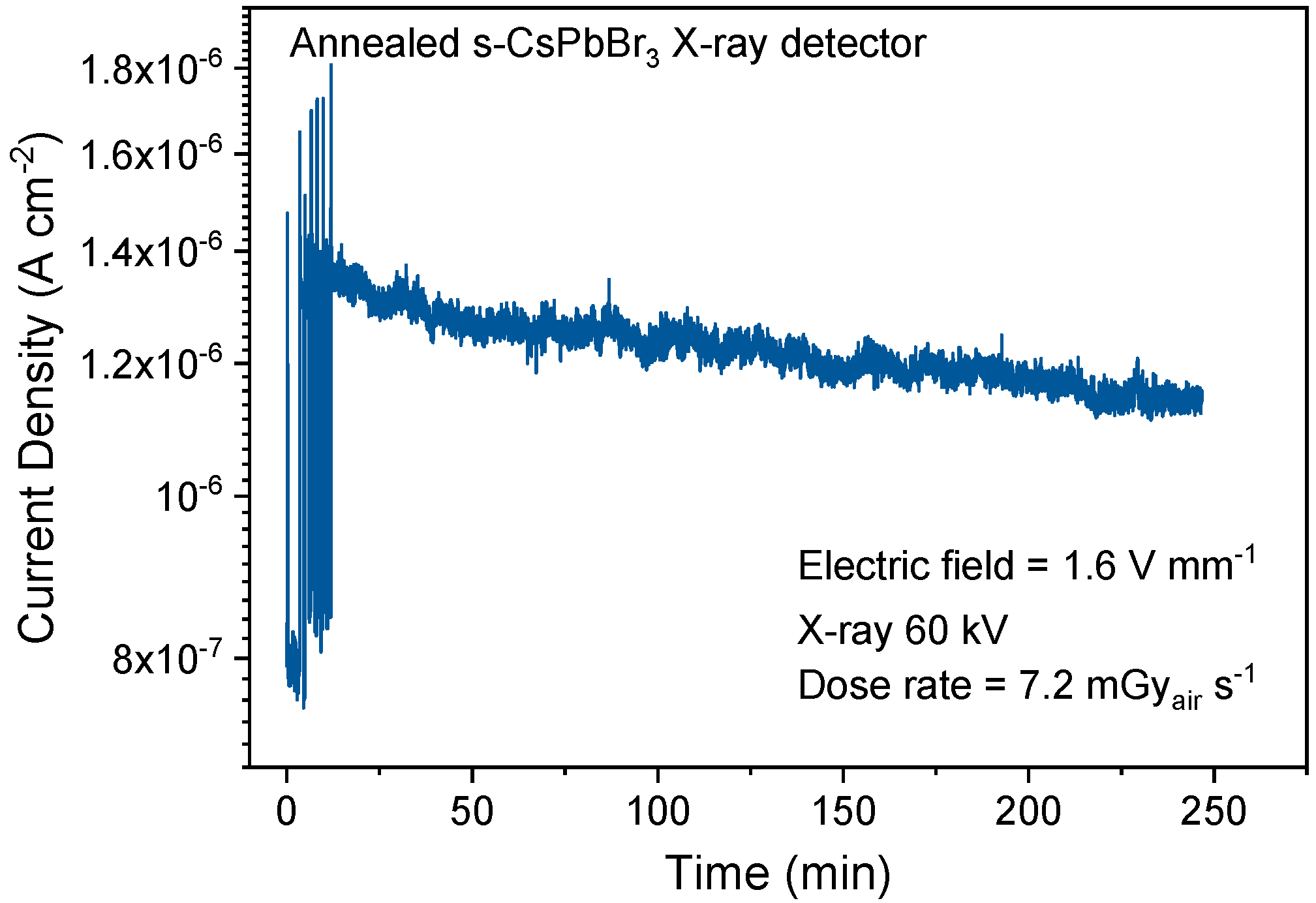


**Figure S11.** Stability of the annealed s-CsPbBr_3_ X-ray detector under continuous X-ray radiation measured at room temperature (28 ^o^C) and 30% relative humidity.


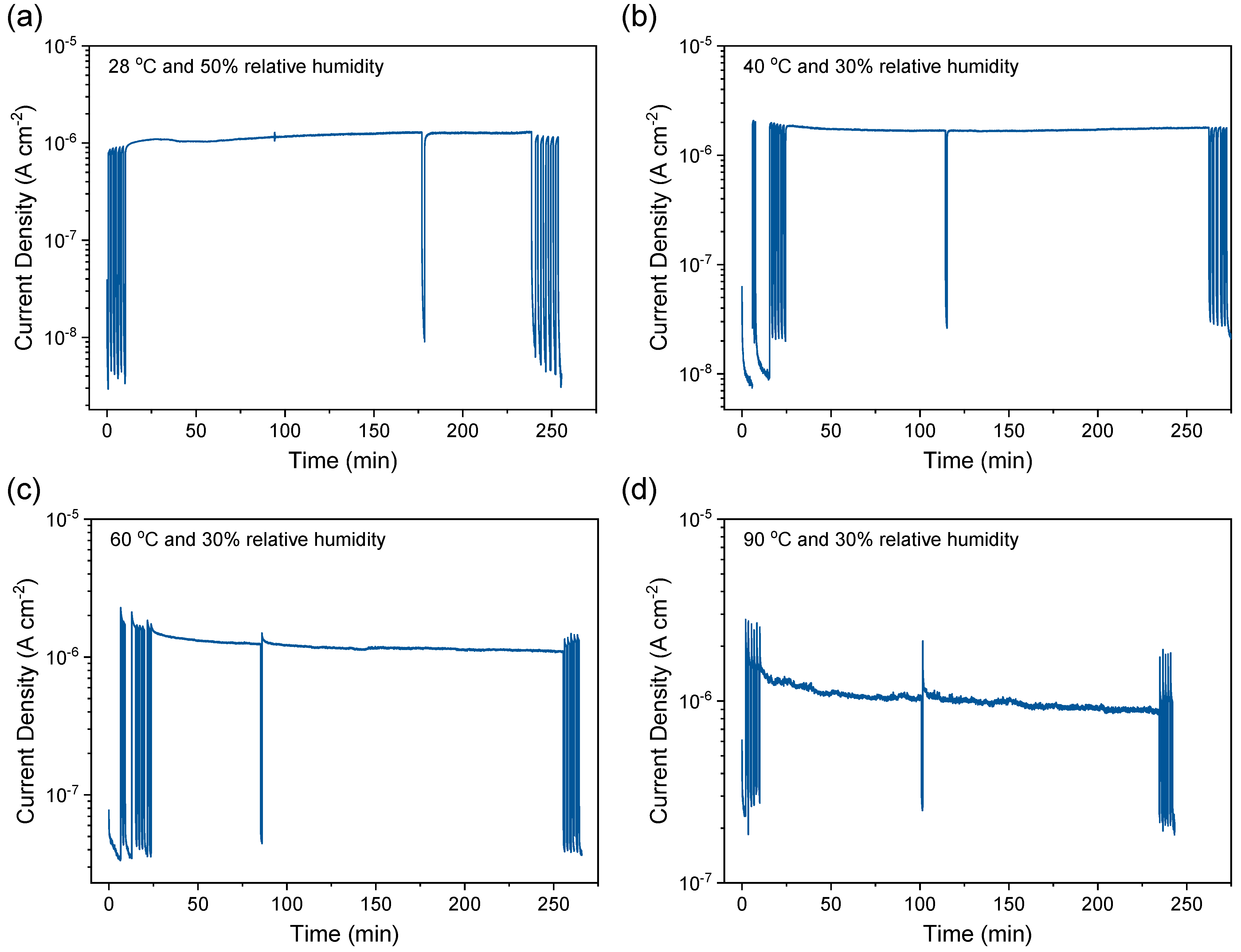


**Figure S12**. Stability of the annealed n-CsPbBr_3_ X-ray detector measured at various temperatures and humidities including several on/off responses under X-ray irradiation. (a) 28 ^o^C and 50 % relative humidity. (b) 40 ^o^C and 30 % relative humidity. (c) 60 ^o^C and 30 % relative humidity. (d) 90 ^o^C and 30 % relative humidity.


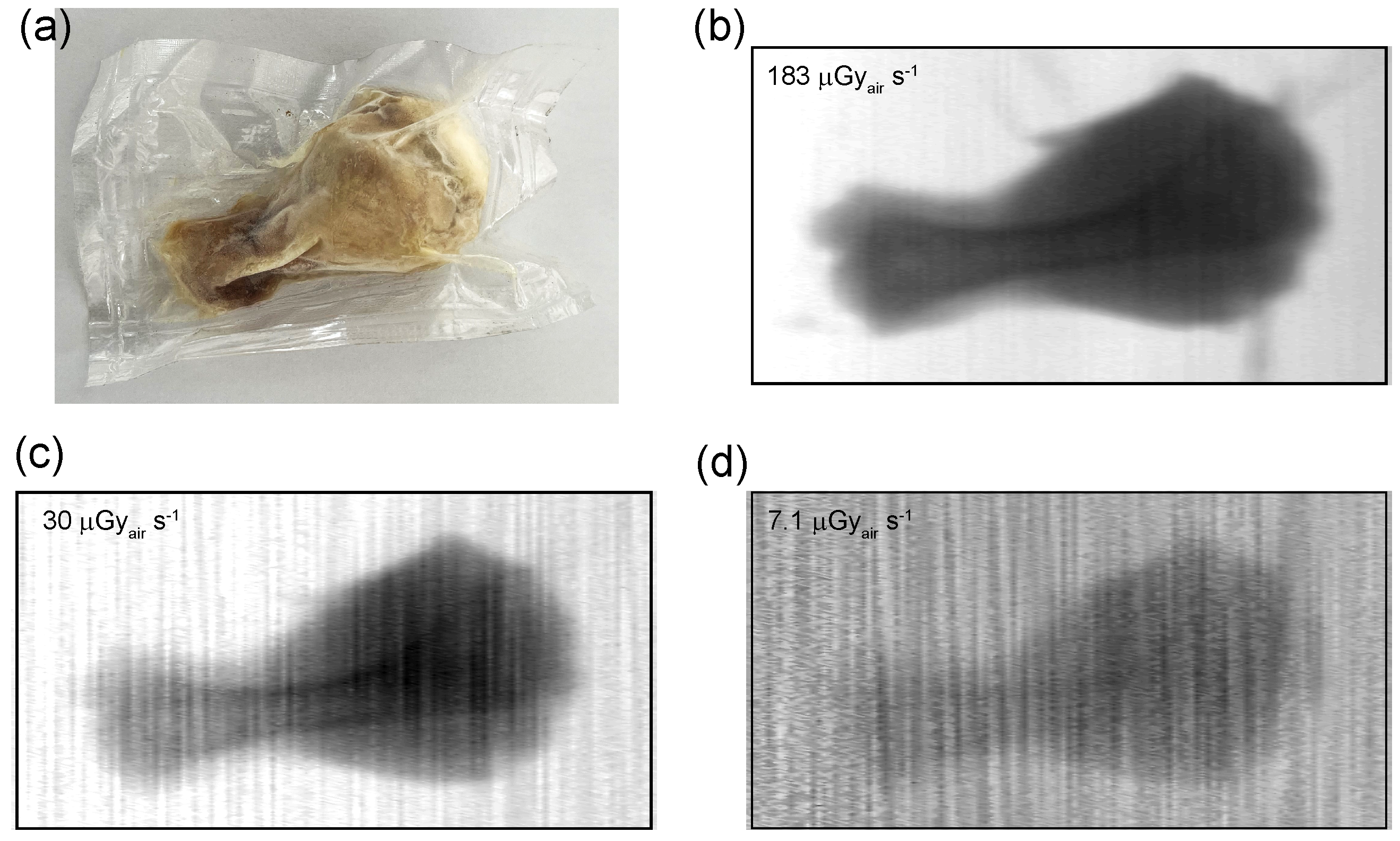


**Figure S13.** X-ray imaging. (a) Digital photo of the chicken drumette used for X-ray imaging. (b-c) X-ray images obtained with a single-pixel detector (1 × 2 mm^2^) by scanning the XY directions at various dose rates: (b) 183 μGy_air_ s^-1^, (c) 30 μGy_air_ s^-1^, and (d) 7.1 μGy_air_ s^-1^.


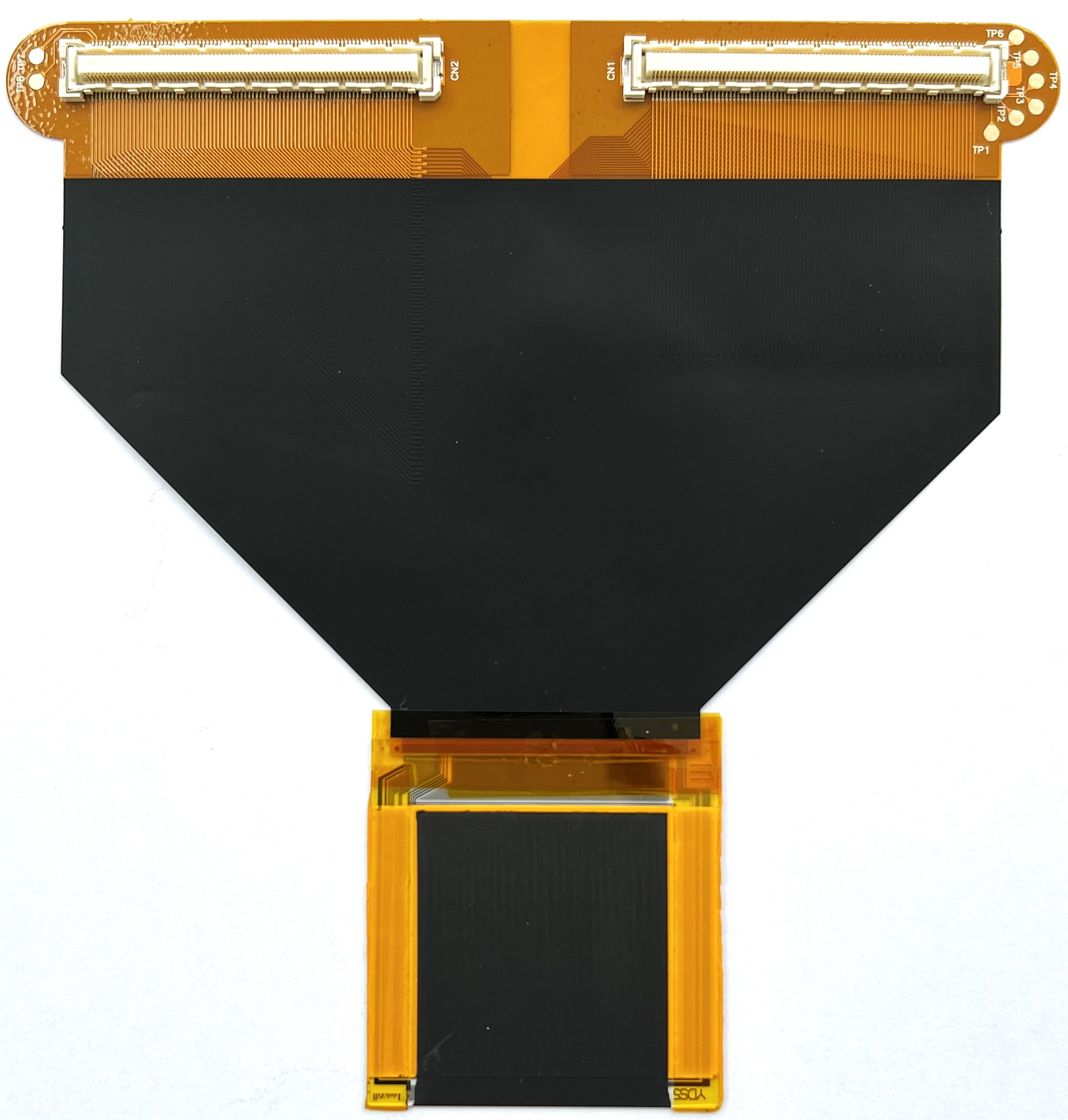


**Figure S14.** Integrated CsPbBr_3_ FPD after blading the carbon electrode and bonding with a flexible printed circuit (FPC).


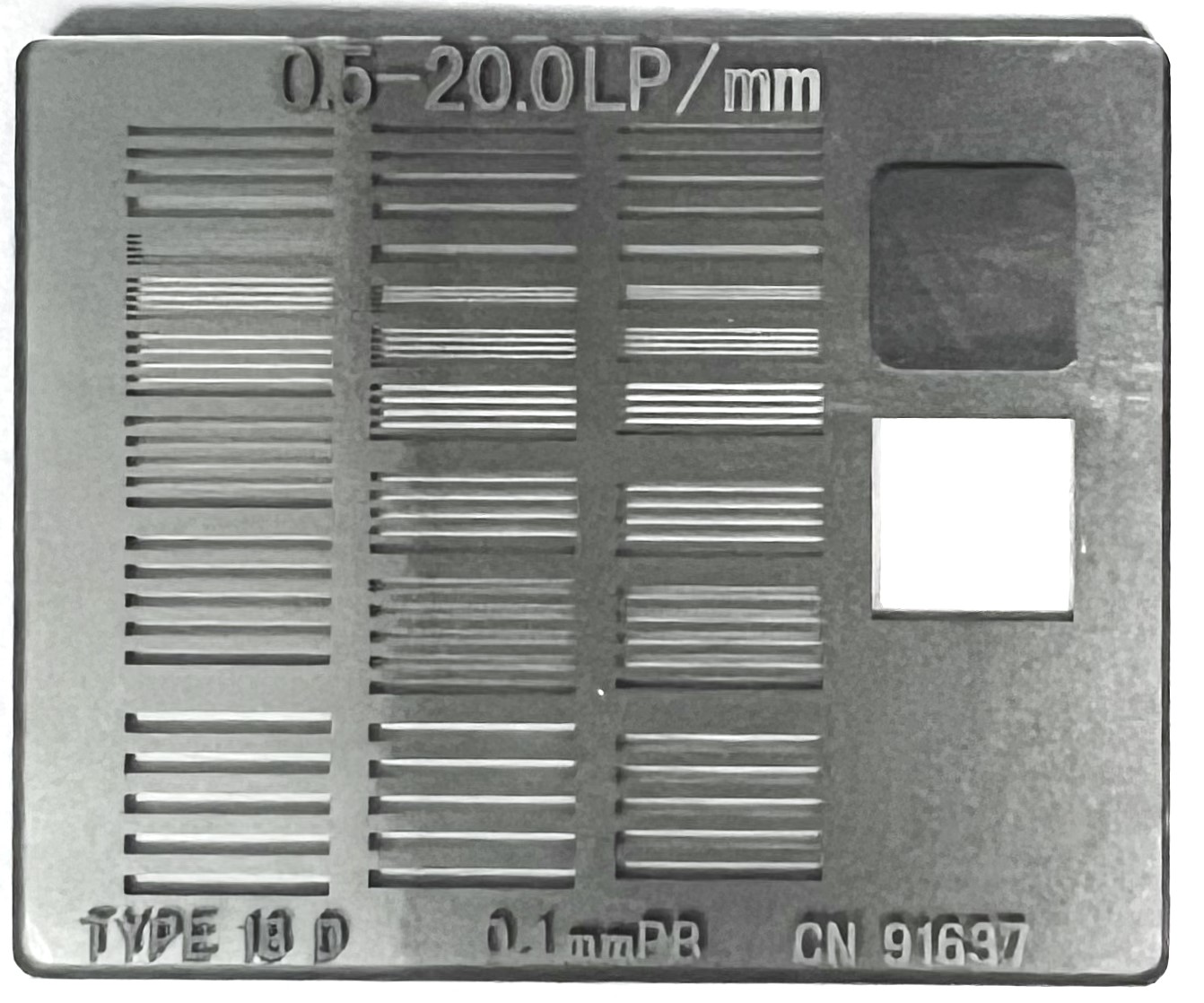


**Figure S15.** Photo of a standard line pair pattern (Model: Type 18 D, QUART GmbH, Germany) used to determine the resolution.


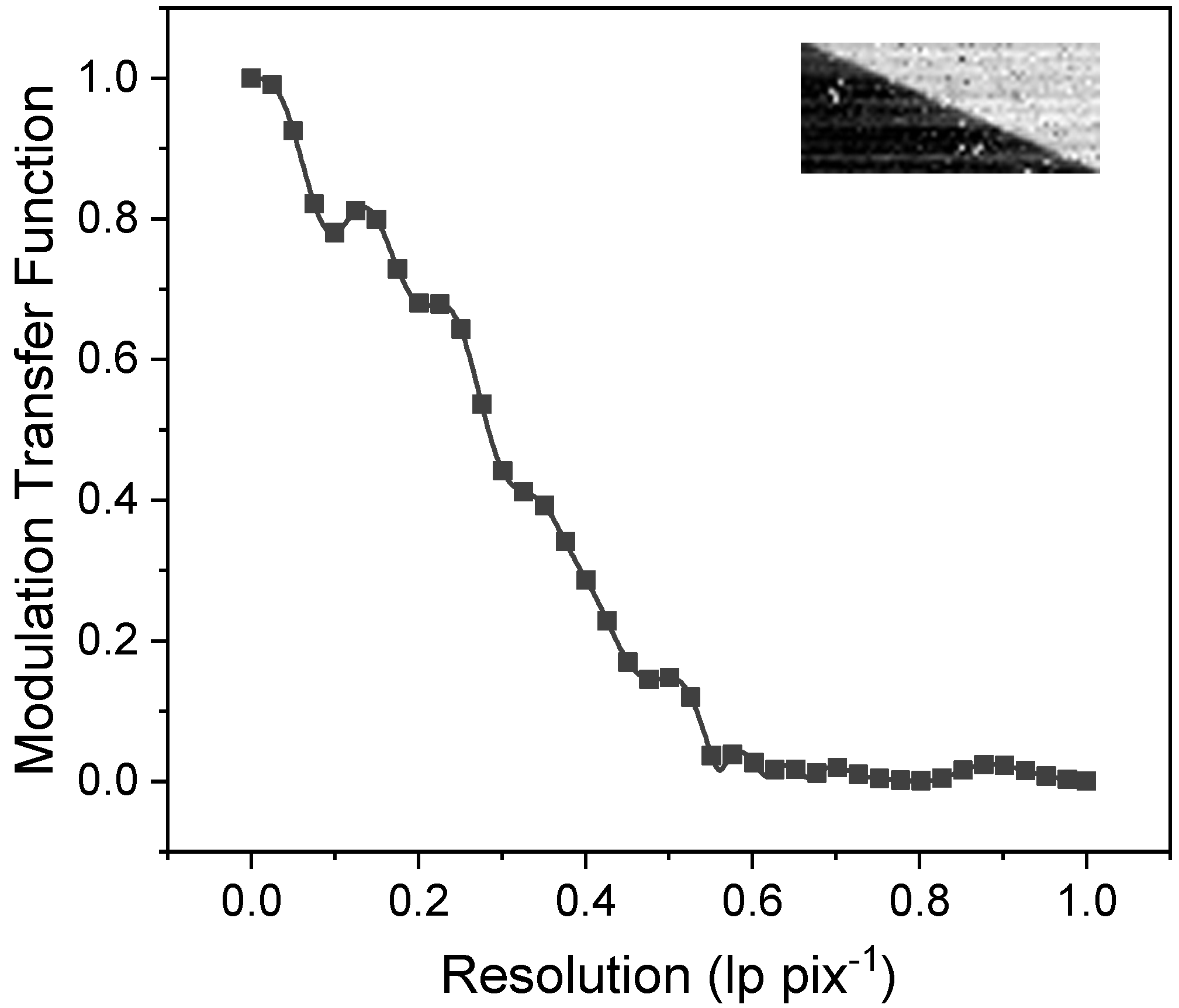


**Figure S16.** MTF curve of the X-ray FPD measured with a 1 mm plastic sheet placed between tungsten and the detector. The resolution is 0.44 lp pix^-1^ at MTF = 0.2, which is equivalent to 3.5 lp mm^-1^ considering a pixel size of 126 μm.


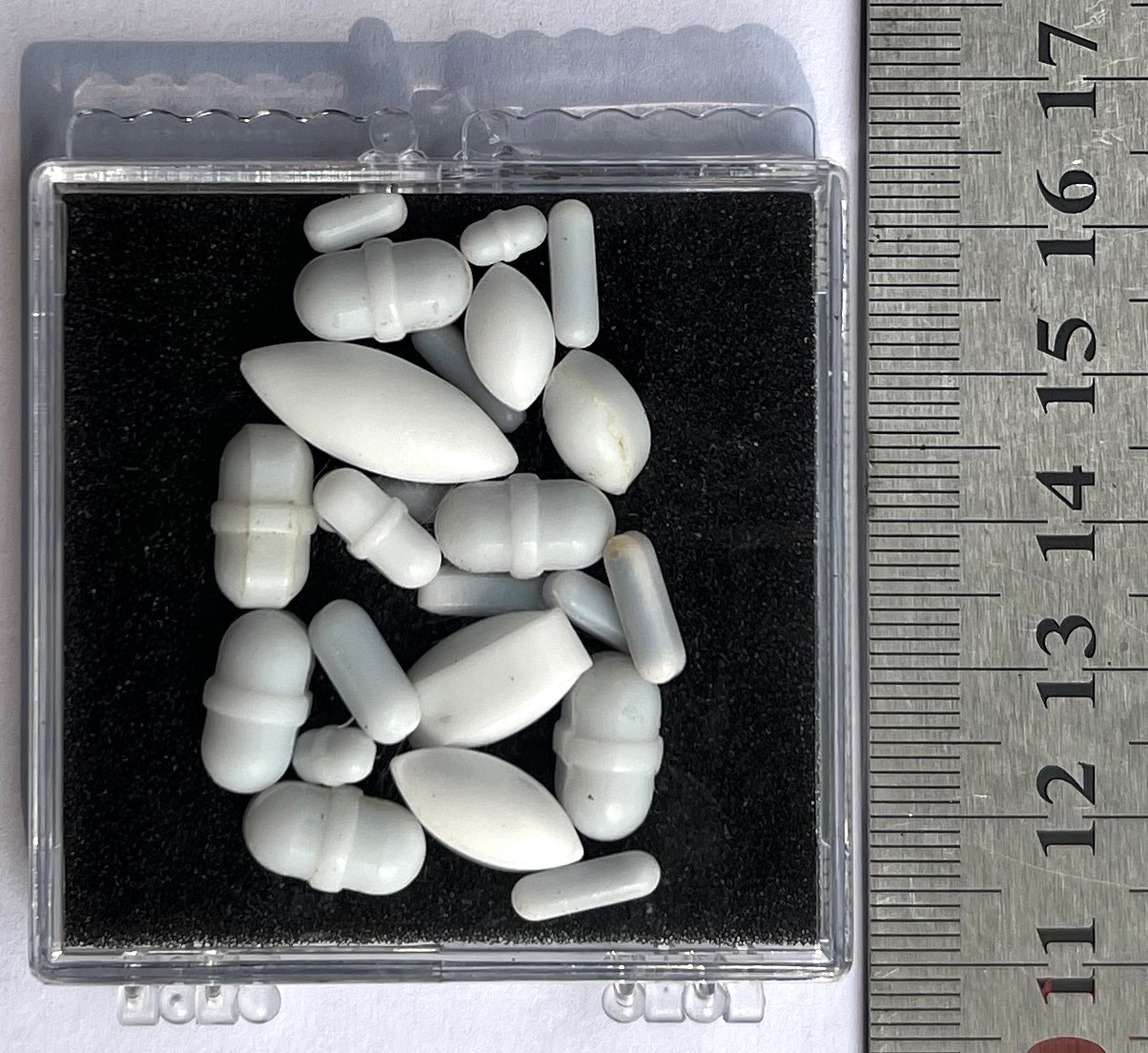


**Figure S17.** A plastic box of magnetic polytetrafluoroethylene (PTFE) stir bars with various sizes used for X-ray imaging.

**Table S1.** Performance comparison of CsPbBr_3_-based X-ray detectors.

| Active layer | Device structure | μτ product  (cm^2^ V^-1^) | Thickness and bias voltage | kVp for X-ray | Dark current density  (A cm^-2^) | Sensitivity  (μC Gy_air_^-1^ cm^-2^) | Limit of detection (LoD, nGy_air_ s^-1^) | Ref. |  |
| --- | --- | --- | --- | --- | --- | --- | --- | --- | --- |
| CsPbBr_3_ wafer | Au/PCBM/CsPbBr_3_ wafer/Au | 5.16 × 10^-4^ | 100 V mm^-1^  40 V mm^-1^  (300- 600 μm) | 50 kV | ~2.0 × 10^-6^ @ 40 V mm^-1^ | 14430  2640 | 564 | [3] |  |
| CsPbBr_3_/CsPb_2_Br_5_ wafer | Au/CsPbBr_3_ wafer/Au | 3.38 × 10^-3^ | 25 V mm^-1^  (1 mm) | 50 kV | ~70.0 × 10^-6^ | 2.58 × 10^5^ | 127.9 | [4] |  |
| CsPbBr_3_ polycrystalline film | ITO/SnO_2_/CsPbBr_3_/Carbon | 5.2 × 10^-4^ | 80 V mm^-1^ | 50 kV | ~5.0 × 10^-6^ | 1.6 × 10^4^ | 102 @ 40 V mm^-1^  321 @ 80 V mm^-1^ | [5] |  |
| CsPbBr_3_ single crystal | Bi/ZnO/C_60_/CsPbBr_3_/C_60_/NiO_x_/Bi | Electron: 2.05 × 10^-3^  Hole: 0.58 × 10^-3^ | 50 V mm^-1^  (2 mm) | 70 kV | ~1.66 × 10^-6^ | 396 |  | [6] |  |
| CsPbBr_3_ single crystal | ITO/NiO_x_/Poly-TPD/CsPbBr_3_/C_70_/BCP/Cu | Electron: 2.69 × 10^-6^  Hole: 2.15 × 10^-6^  (Calculated) | 5 V mm^-1^  (~1 mm) | 60 kV |  | 4086 |  | [7] |  |
| CsPbBr_3_ polycrystalline film | | Tungsten (W)/CsPbBr_3_/Polymer/Pt |  | 45.5 V mm^-1^  （110 μm） | 70 kV | 0.28 × 10^-6^ | 11840 |  | [8] |
| CsPbBr_3_ polycrystalline film | | FTO/CsPbBr_3_/Au | 1.32 × 10^-2^ | 5.0 V mm^-1^  (240 μm) | 30 kV |  | 55684 | 215 | [9] |
| CsPbBr_3_ polycrystalline film | | ITO/CsPbBr_3_/Au |  | 0 V mm^-1^  110 V mm^-1^  (18 μm) | 35 kV |  | 470  1700 | 53 | [10] |
| CsPbBr_3_ polycrystalline film | FTO/CsPbBr_3_/Carbon | 3.0 × 10^-5^ | 1.6 V mm^-1^  40 V mm^-1^  (25 μm) | 60 kV | 2.8 × 10^-9^  4.6 × 10^-6^ | 153.0  4.2 × 10^4^ | 136 @ 1.6 V mm^-1^ | This work |  |

**Table S2.** Comparison of perovskite flat-panel X-ray detectors.

| Active layer | Backplane | Pixel pitch | Pixel count | Detection area | Resolution | Ref. |
| --- | --- | --- | --- | --- | --- | --- |
| MAPbI_3_ | a-Si:H TFT  (iRay) | 150 μm | 256 × 256 | 3.84 × 3.84 cm^2^ | 0.51 lp pix^-1^ @ MTF = 0.2  3.4 lp mm^-1^ @MTF = 0.2 | [11] |
| MAPbI_3_ | a-Si:H TFT  (CMO Japan) | 70 μm | 1428 × 1428 | 10 × 10 cm^2^ | 0.217 lp pix^-1^ @ MTF = 0.2  3.1 lp mm^-1^ @ MTF = 0.2 | [12] |
| MAPbI_3_ | IGZO TFT  (Holst Centre) | 50 μm | 640 × 480 | 3.2 × 2.4 cm^2^ | 0.165 lp pix^-1^ @ MTF = 0.2  3.3 lp mm^-1^ @ MTF = 0.2 | [13] |
| CsPbBr_3_/CsPb_2_Br_5_ | a-Si:H TFT  (LinkZill) | 500 μm | 64 × 64 | 3.2 × 3.2 cm^2^ | 0.785 lp pix^-1^ @ MTF = 0.2  1.57 lp mm^-1^ @ MTF = 0.2 | [4] |
| CsPbBr_3_ | CMOS  (Home-made) | 83.2 μm | 72 × 72 | 0.6 × 0.6 cm^2^ | 0.416 lp mm^-1^ @ MTF = 0.1  5.0 lp mm^-1^ @ MTF = 0.1 | [5] |
| MAPbI_3_ | a-Si:H TFT  (LinkZill) | 200 μm | 64 × 64 | 1.28 × 1.28 cm^2^ | 0.52 lp pix^-1^ @ MTF = 0.2  2.6 lp mm^-1^ @ MTF = 0.2 | [14] |
| FA_0.9_MA_0.05_Cs_0.05_Pb(I_0.9_Br_0.1_)_3_ | a-Si:H TFT  (LinkZill) | 500 μm | 64 × 64 | 3.2 × 3.2 cm^2^ | 0.46 lp pix^-1^ @ MTF = 0.2  0.92 lp mm^-1^ @ MTF = 0.2 | [15] |
| CsPbBr_3_ | IGZO TFT  (LinkZill) | 126 μm | 256 × 256 | 3.2 × 3.2 cm^2^ | 0.65 lp pix^-1^ @ MTF = 0.2  5.2 lp mm^-1^ @ MTF = 0.2 | This work |

**Table S3.** Comparison with conventional flat-panel X-ray detectors.

| Materials | Sensitivity  (μC Gy_air_^-1^ cm^-2^) | Limit of detection(LoD, nGy_air_ s^-1^) | Resolution  (lp mm^-1^@ MTF = 0.2) | Fabrication Process | Cost | Ref. |
| --- | --- | --- | --- | --- | --- | --- |
| CsI (Tl) | 5370 | 180 | 10 | Vacuum Thermal Evaporation | Moderate-cost | [16] |
| GOS (Tb) | 7.3 |  | 4.75 | High-Temperature Solid Phase Method | Low-cost | [17] |
| Si | 8 | 8.3×10^6^ | 4.5 | Single crystal | High-cost | [18] |
| CZT | 318 | 5×10^4^ | 10 | Single crystal | High-cost | [19] |
| α-Se | 20 | 5.5×10^3^ | 15 | Chemical Vapor Deposition | Moderate-cost | [20, 21] |
| HgI_2_ | 1600 | 1×10^4^ | 3.93 | Physical Vapor Deposition | Moderate-cost | [22] |
| CsPbBr_3_ | 4.2 × 10^4^ | 138 | 5.2 | ALS method | Low-cost | This work |

References:

[1] B. Fraboni, A. Ciavatti, F. Merlo, L. Pasquini, A. Cavallini, A. Quaranta, A. Bonfiglio, A. Fraleoni-Morgera, *Adv. Mater.* **2012**, *24*, 2289.

[2] X. Xu, W. Qian, J. Wang, J. Yang, J. Chen, S. Xiao, Y. Ge, S. Yang, *Adv Sci (Weinh)* **2021**, *8*, e2102730.

[3] T. Y. Shi, W. J. Liu, J. T. Zhu, X. S. Fan, Z. Y. Zhang, X. C. He, R. He, J. H. Wang, K. Z. Chen, Y. S. Ge, X. M. Sun, Y. L. Liu, P. K. Chu, X. F. Yu, *Nano Res.* **2023**, *16*, 9983.

[4] C. Wan, Z. Wang, H. Zhang, S. Tie, Z. Liang, H. Xu, Y. Ma, Z. Wang, X. Zheng, X. Pan, J. Ye, *Adv. Funct. Mater.* **2024**.

[5] Y. Liu, C. Gao, D. Li, X. Zhang, J. Zhu, M. Wu, W. Liu, T. Shi, X. He, J. Wang, H. Huang, Z. Sheng, D. Liang, X.-F. Yu, H. Zheng, X. Sun, Y. Ge, *Nat. Commun.* **2024**, *15*, 1588.

[6] J. Pang, H. Wu, H. Li, T. Jin, J. Tang, G. Niu, *Nat. Commun.* **2024**, *15*, 1769.

[7] J. Peng, C. Q. Xia, Y. Xu, R. Li, L. Cui, J. K. Clegg, L. M. Herz, M. B. Johnston, Q. Lin, *Nat. Commun.* **2021**, *12*, 1531.

[8] Y. Haruta, T. Ikenoue, M. Miyake, T. Hirato, *MRS Adv.* **2020**, *5*, 395.

[9] W. Pan, B. Yang, G. Niu, K. H. Xue, X. Du, L. Yin, M. Zhang, H. Wu, X. S. Miao, J. Tang, *Adv. Mater.* **2019**, *31*, 1904405.

[10] Z. Gou, S. Huanglong, W. Ke, H. Sun, H. Tian, X. Gao, X. Zhu, D. Yang, P. Wangyang, *physica status solidi (RRL) – Rapid Research Letters* **2019**, *13*, 1900094.

[11] Z. Song, X. Du, X. He, H. Wang, Z. Liu, H. Wu, H. Luo, L. Jin, L. Xu, Z. Zheng, G. Niu, J. Tang, *Nat. Commun.* **2023**, *14*, 6865.

[12] Y. C. Kim, K. H. Kim, D. Y. Son, D. N. Jeong, J. Y. Seo, Y. S. Choi, I. T. Han, S. Y. Lee, N. G. Park, *Nature* **2017**, *550*, 87.

[13] S. Deumel, A. Van Breemen, G. Gelinck, B. Peeters, J. Maas, R. Verbeek, S. Shanmugam, H. Akkerman, E. Meulenkamp, J. E. Huerdler, M. Acharya, M. García-Batlle, O. Almora, A. Guerrero, G. Garcia-Belmonte, W. Heiss, O. Schmidt, S. F. Tedde, *Nat. Electron.* **2021**, *4*, 681.

[14] Z. Liu, H. Wu, H. Yang, Z. Song, X. Du, X. He, W. Xiang, Y. Shi, J. Pang, L. Xu, Z. Zheng, Y. Zhang, J. Tang, G. Niu, *ACS Energy Lett.* **2024**, 1397.

[15] H. Wu, X. Chen, Z. Song, A. Zhang, X. Du, X. He, H. Wang, L. Xu, Z. Zheng, G. Niu, J. Tang, *Adv. Mater.* **2023**.

[16] D. J. Starkenburg, P. M. Johns, J. E. Baciak, J. C. Nino, J. Xue, *J. Appl. Phys.* **2017**, *122*.

[17] P. Büchele, M. Richter, S. F. Tedde, G. J. Matt, G. N. Ankah, R. Fischer, M. Biele, W. Metzger, S. Lilliu, O. Bikondoa, J. E. Macdonald, C. J. Brabec, T. Kraus, U. Lemmer, O. Schmidt, *Nat. Photonics* **2015**, *9*, 843.

[18] G. Rikner, E. Grusell, *Phys. Med. Biol.* **1983**, *28*, 1261.

[19] H. Wei, J. Huang, *Nat. Commun.* **2019**, *10*, 1066.

[20] S. O. Kasap, *J. Phys. D Appl. Phys.* **2000**, *33*, 2853.

[21] S. Kasap, J. B. Frey, G. Belev, O. Tousignant, H. Mani, J. Greenspan, L. Laperriere, O. Bubon, A. Reznik, G. DeCrescenzo, K. S. Karim, J. A. Rowlands, *Sensors* **2011**, *11*, 5112.

[22] G. Zentai, L. Partain, R. Pavlyuchkova, C. Proano, G. Virshup, L. Melekhov, A. Zuck, B. Breen, O. Dagan, A. Vilensky, M. Schieber, H. Gilboa, P. Bennet, K. Shah, Y. Dmitriyev, J. Thomas, M. Yaffe, D. Hunter, *Mercuric iodide and lead iodide x-ray detectors for radiographic and fluoroscopic medical imaging, Vol. 5030*, SPIE, **2003**.
